# Supplementary material for: Flow driven robotic navigation of microengineered endovascular probes
Source: Nat Commun. 2020 Dec 22;11:6356. doi: 10.1038/s41467-020-20195-z (PMC7755921; doi:10.1038/s41467-020-20195-z)
Supplement: Supplementary file 1 — Supplementary Information [file 41467_2020_20195_MOESM1_ESM.pdf]

Supplementary Information

Flow-driven robotic navigation of microengineered endovascular probes

Lucio Pancaldi[1], Pietro Dirix[1], Adele Fanelli[2], Augusto Martins Lima[3], Nikolaos Stergiopulos[3], Pascal John Mosimann[4], Diego Ghezzi[2] and Mahmut Selman Sakar[1,3*]

[1] Institute of Mechanical Engineering, Ecole Polytechnique Fédérale de Lausanne (EPFL), 1015 Lausanne, Switzerland

[2] Medtronic Chair in Neuroengineering, Center for Neuroprosthetics and Institute of Bioengineering, School of Engineering, EPFL, 1202 Geneva, Switzerland

[3] Institute of Bioengineering, EPFL, 1015 Lausanne, Switzerland

[4] Institute for Diagnostic and Interventional Neuroradiology, 3010 Bern, Switzerland

[5] Department of Diagnostic and Interventional Neuroradiology, Alfried Krupp Krankenhaus, 45130 Essen, Germany

* Corresponding author: selman.sakar@epfl.ch

Supplementary Figures

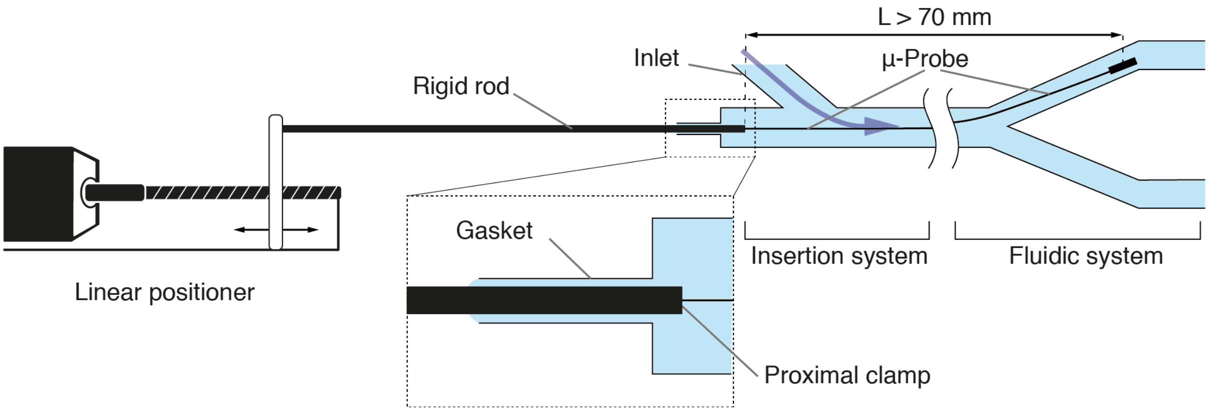

Supplementary Figure 1. Illustration showing the insertion system. The μ-probe is proximally coupled to a rigid rod and its position controlled by the movement of the rod. The main purpose of the rigid rod is to provide a rigid coupling across the gasket between the linear positioner and the flexible μ-probe, and to control the release rate of the μ-probe that is being pulled by the flow. The μ-probes used in this study were longer than 70 mm. Drawings are not to scale.

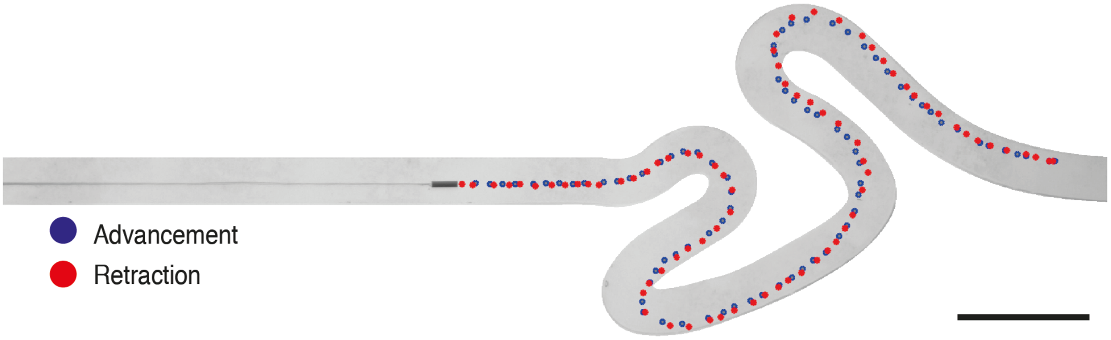

Supplementary Figure 2. Visualization of the tip trajectories during advancement and retraction of a μ-probe. The tip of the μ-probe follows a very similar path during advancement (blue dots) and retraction (red dots). Scale bar, 5 mm.

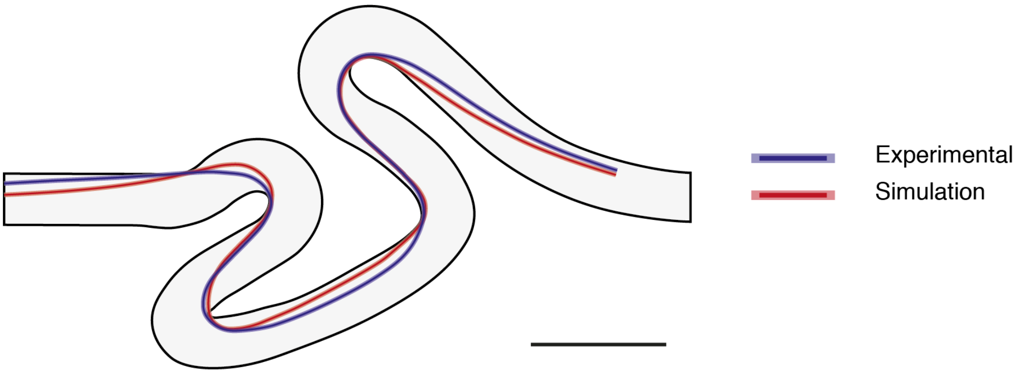

Supplementary Figure 3. Comparative analysis of empirical and numerical results. Comparison between the steady-state shape of the μ-probe in experimental (blue) and simulation (red). Scale bar, 5 mm.

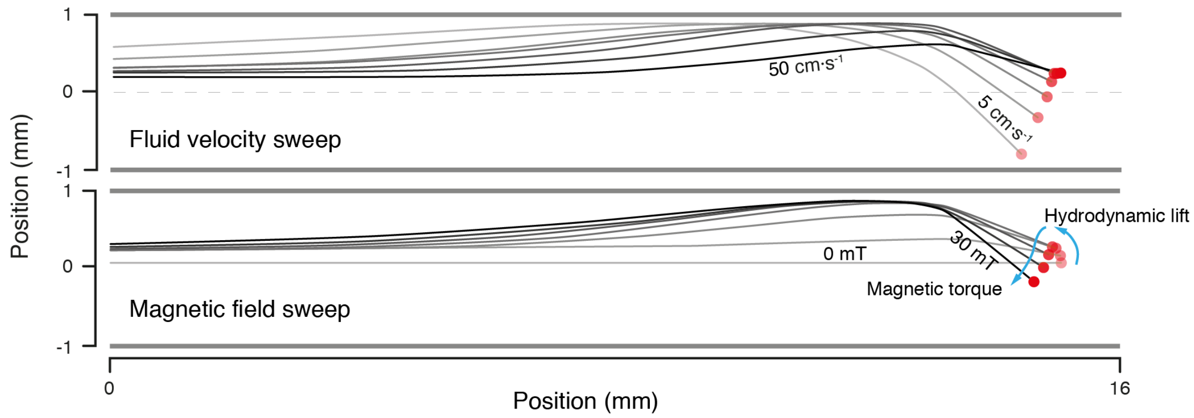

Supplementary Figure 4. The influence of magnetic field and flow velocity on the equilibrium pose. Skeleton overlays of a μ-probe subjected to varying flow velocity (top) and magnetic torque (bottom). The magnetic field strength was set to 8 mT for the velocity sweep and the flow velocity was kept at 30 cm·s^{-1} for the magnetic field sweep. Red dots indicate the position of the tip.

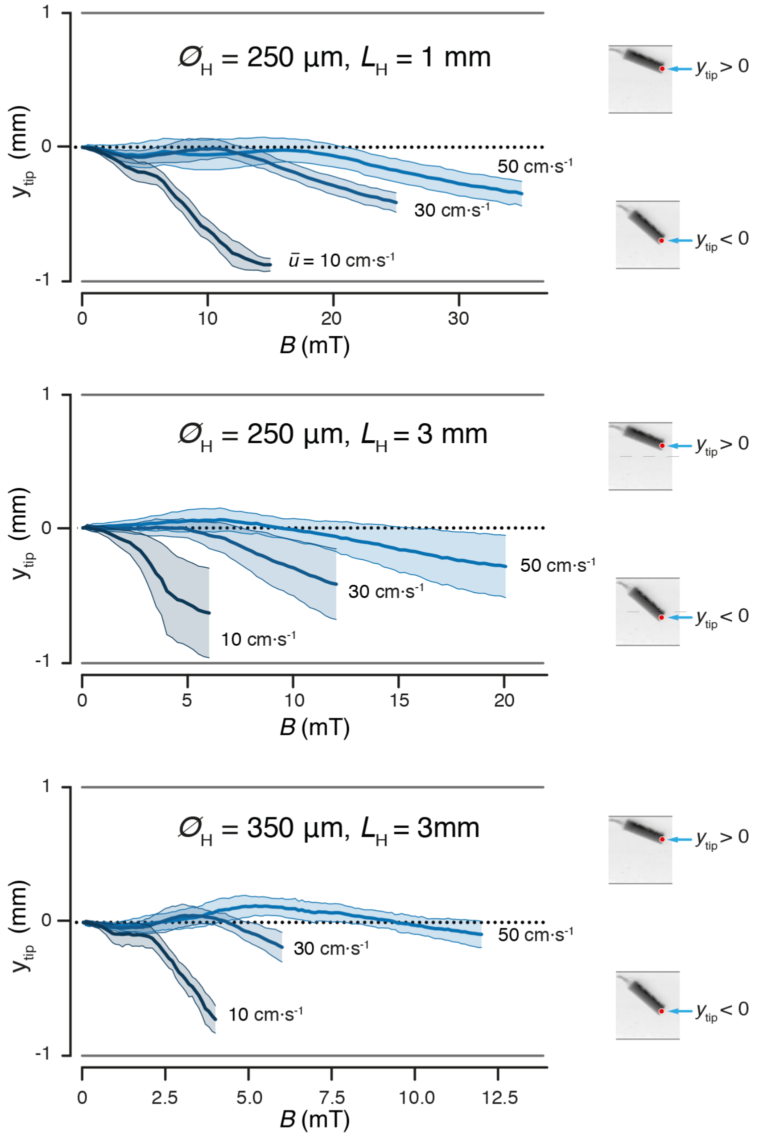

Supplementary Figure 5. The influence of magnetic head geometry on the equilibrium pose. The displacement of the tip for three different magnetic head geometries at varying magnetic fields and flow velocities. Darker lines represent the average and lighter areas represent the ± standard deviation.

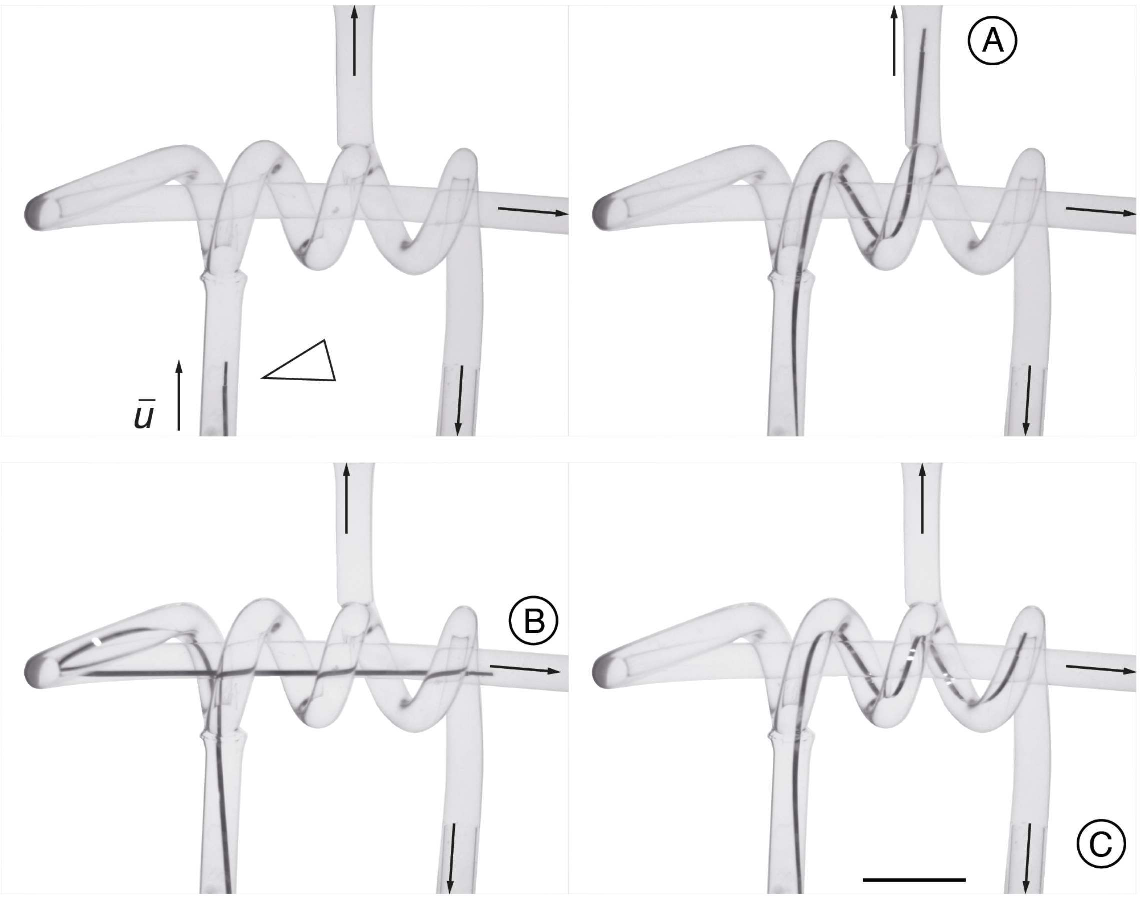

Supplementary Figure 6. Controlled navigation in a 3D channel. The channel is looped around itself and has two vertically diverging bifurcations. The μ-probe successfully reached all 3 target locations (A, B and C) under the guidance of magnetic actuation. The μ-probe was transported at a velocity of 2 cm·s^{-1} at $\bar{u}$ = 30 cm·s^{-1}. Scale bar, 5 mm.

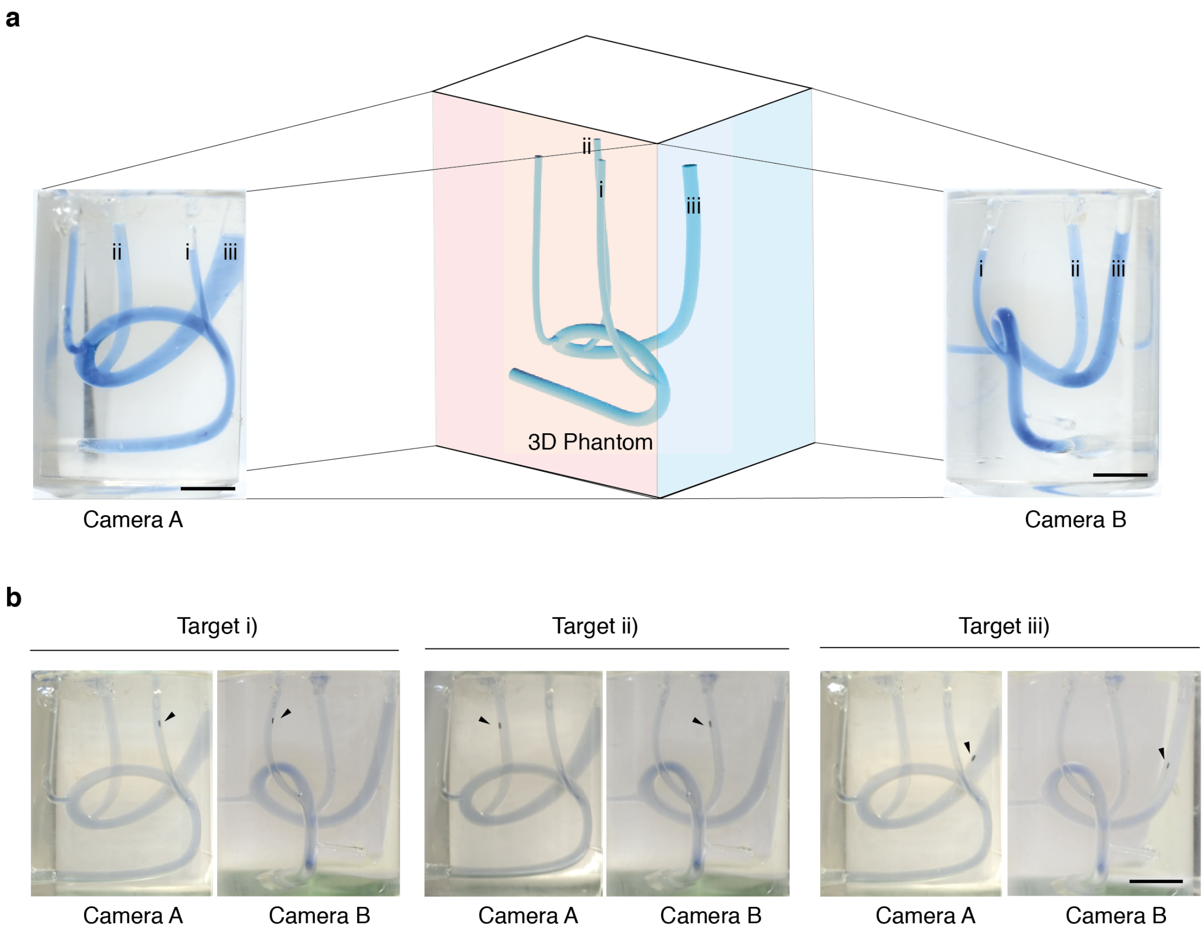

Supplementary Figure 7. Teleoperated navigation in a 3D phantom. a The workspace is observed by two cameras (A and B) for 3D localization. The targets are marked with roman numerals i, ii and iii. **b** Snapshots from the navigation movie showing successful deployment of the μ-probe at different daughter arteries. Scale bars, 5 mm.

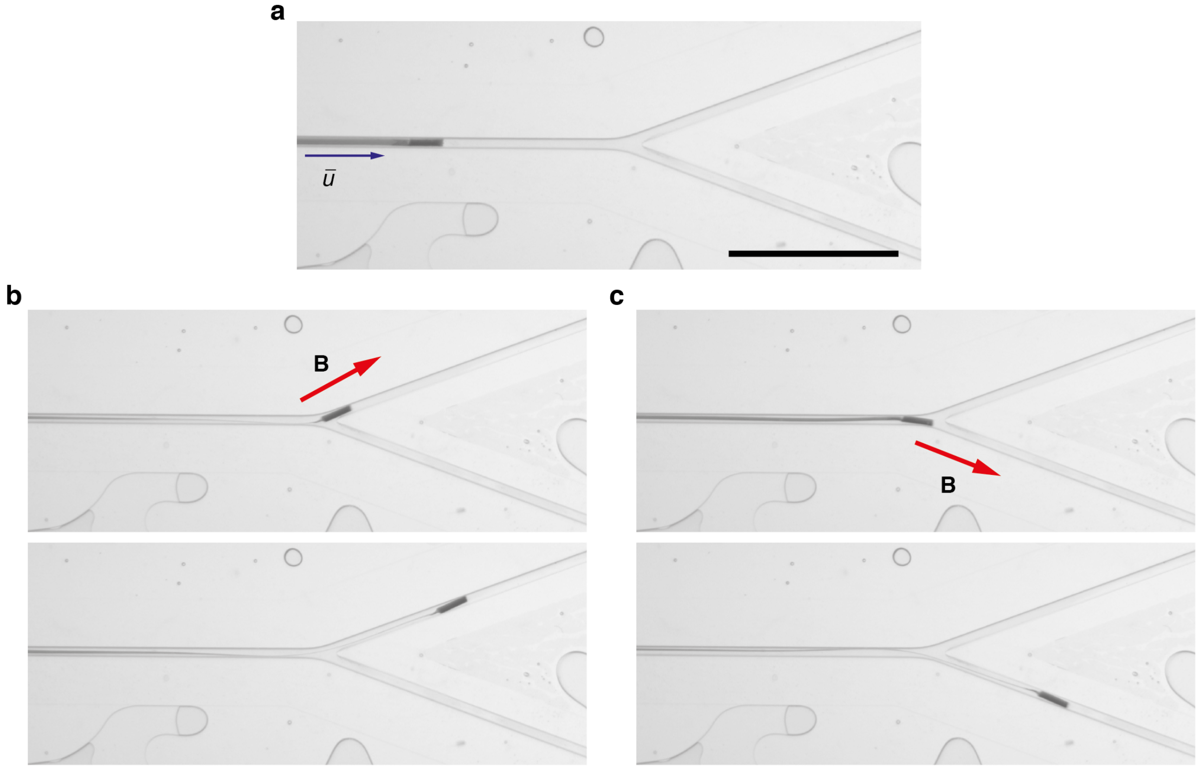

Supplementary Figure 8. Flow-driven navigation in narrow channels. Channel size (300 µm) is comparable to the diameter of the magnetic head diameter (250 µm) at $\bar{u}$ = 0.5 cm·s^{-1}. **a** A µ-probe is deployed into a channel branching into daughter vessels with 20° angle. Scale bar, 5 mm. **b** The magnetic head is steered towards the top channel (top) and released to advance further in the top channel under the control of flow (bottom). **c** The same control strategy is applied to steer the µ-probe into the bottom daughter vessel.

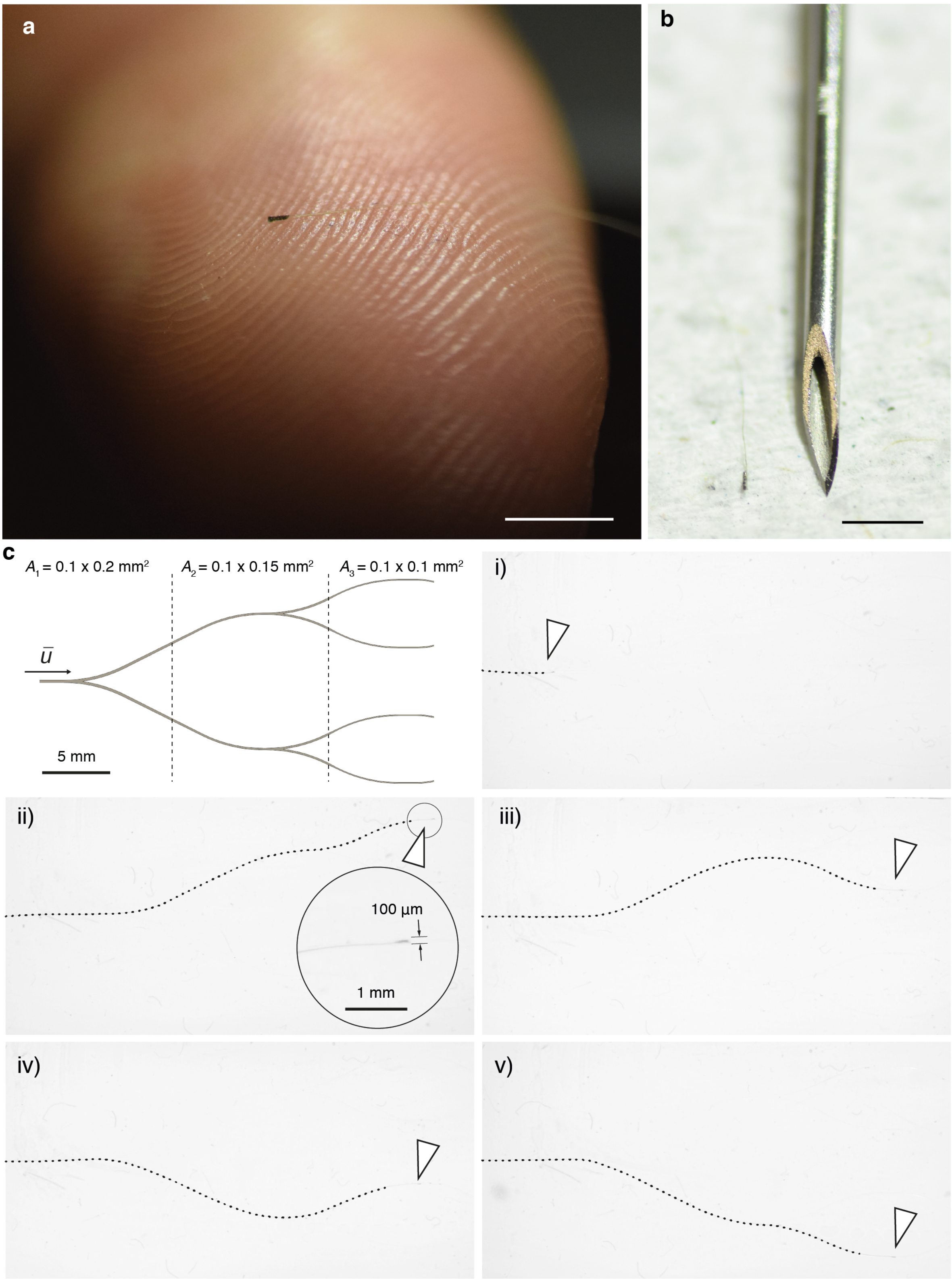

Supplementary Figure 9. Navigation inside microfluidic channels. Picture of a 25 μm-wide ultra-flexible μ-probe (**a**) on a human finger and (**b**) with a 21 G hypodermic needle (0.8 mm diameter). Scale bars, 2 mm **c** Top left figure shows a schematic of the microfluidic channel showing the dimensions of each branch. All channels are 100 μm in height. Upon entry (i) the μ-probe is transported by the fluid and steered with magnetic actuation to reach the target locations shown in (ii-v). Inset in (ii) shows the 25 μm-wide μ-probe and the 40 μm-diameter magnetic head inside the 100 μm x 100 μm channel.

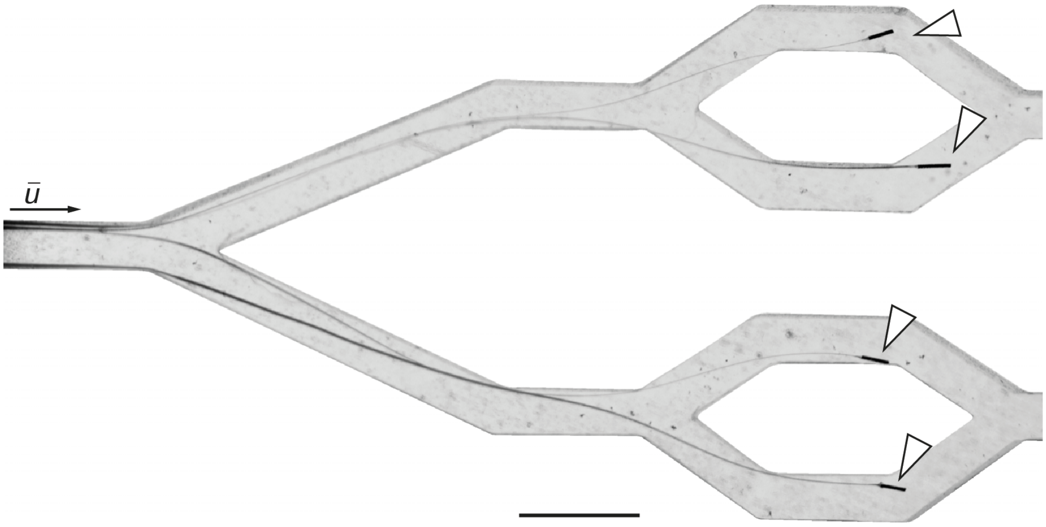

Supplementary Figure 10. Simultaneous deployment of multiple μ-probes. Adaptive and autonomous flow-driven transportation ensures safe passage of the slender structures despite the partial occlusions created by the presence of other μ-probes. Scale bar, 5 mm.

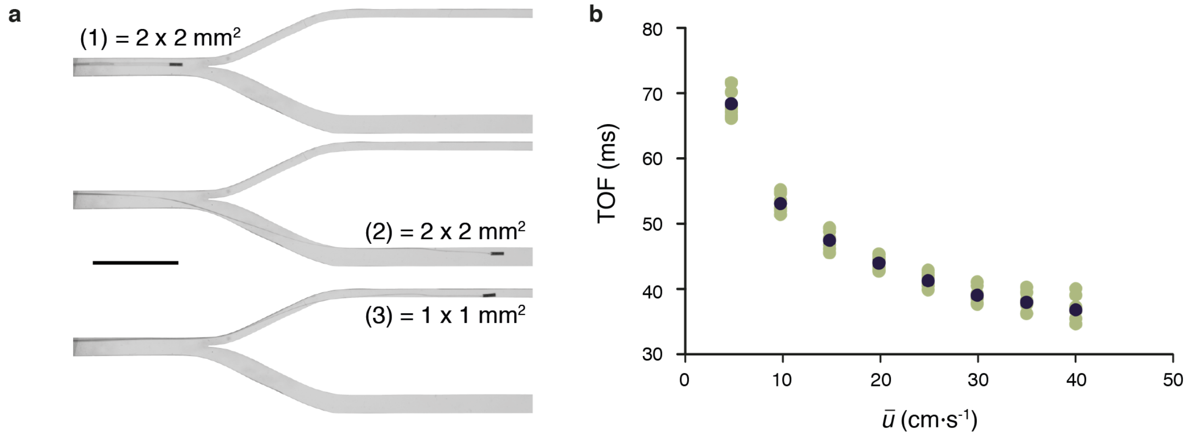

Supplementary Figure 11. Characterization of the flow sensor. a Pictures of the experimental channel where flow measurements were taken. The mother vessel bifurcates into two daughter vessels with different cross-sectional area. Thus, the fluid velocities are expected to be different. Scale bar, 10 mm. **b** The repeatability of the measurements was verified in a 2 mm x 2 mm channel using three different μ-probes and by performing three independent trials with each μ-probe (*n* = 9). Mean value is marked with black dots.

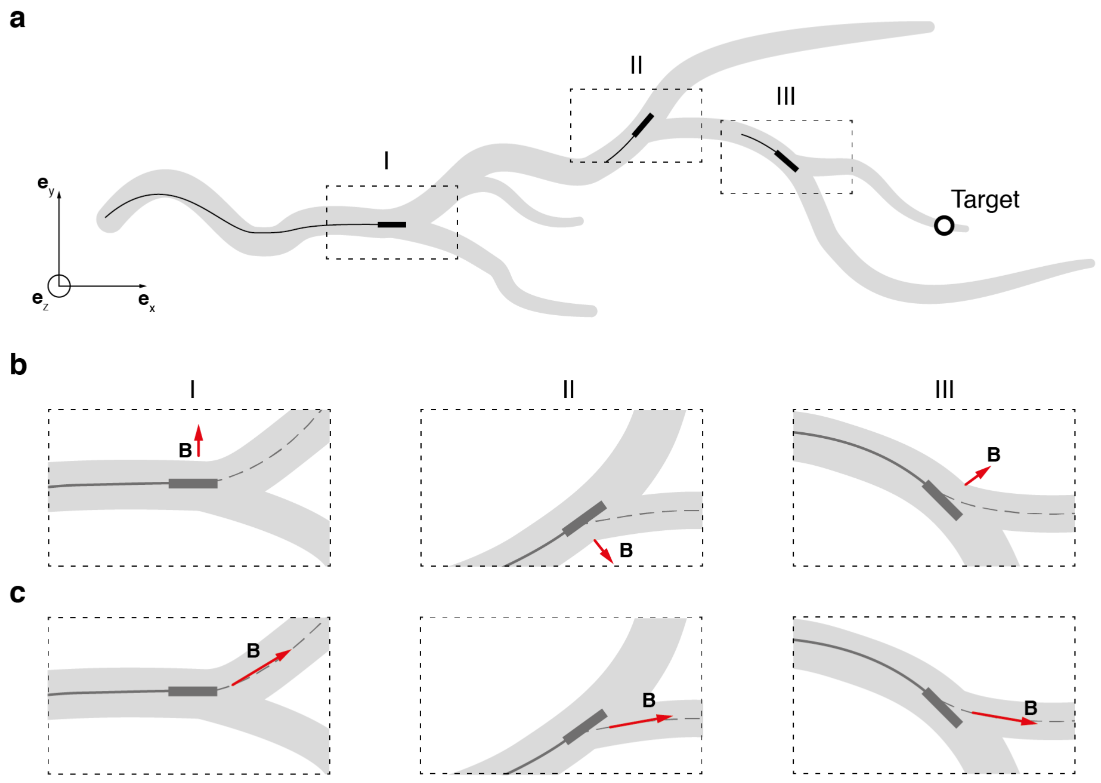

Supplementary Figure 12. 2D projection of proposed 3D navigation control strategies. a Large view of a vascular tree example presenting three bifurcation (I, II, III) leading to the target region (black circle). Fluoroscope views are boxed with black dotted lines. **b** Perpendicular actuation control: the magnetic head is rotated with minimal magnetic field by orienting it perpendicular to the incident vessel (assumed parallel to magnetic head). **c** Target vessel direction control: magnetic field is oriented towards the target vessel and the magnetic field increased until the head is properly oriented. Dark grey dashed lines indicate the target trajectory. Drawings are not to scale.

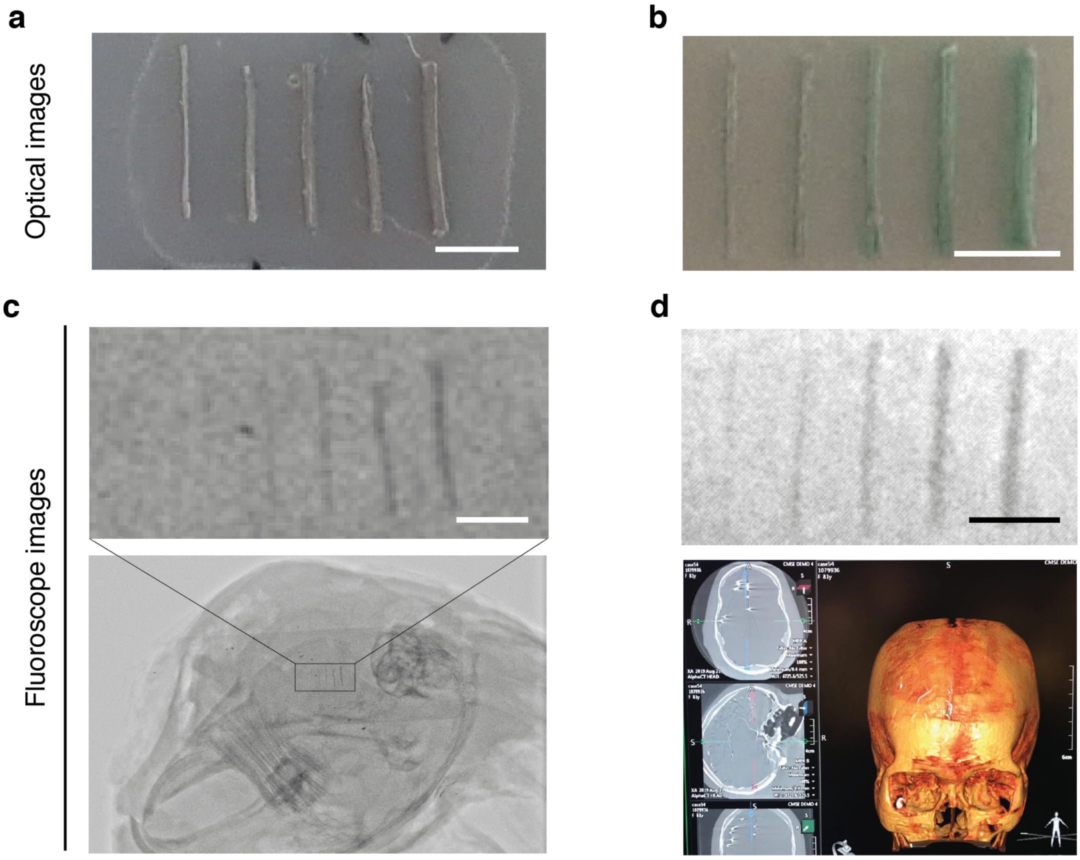

Supplementary Figure 13. **Imaging of μ-probes using two different fluoroscopes in realistic environments. a,b** Optical images of the samples under test. The composite consists of PDMS:NdFeB microparticles that was mixed at a 1:1 volume ratio. The diameter of the magnetic samples varied between 150 μm and 350 μm. **c** Fluoroscope images (Canon Alphenix Sky+, 200 μm resolution) of the magnetic structures (top) placed in an ex vivo rabbit head (bottom). **d** Fluoroscope images (Canon Alphenix Core+, 76 μm resolution) of the magnetic structures (top) inside an anthropomorphic head phantom (bottom). Scale bars, 5 mm.

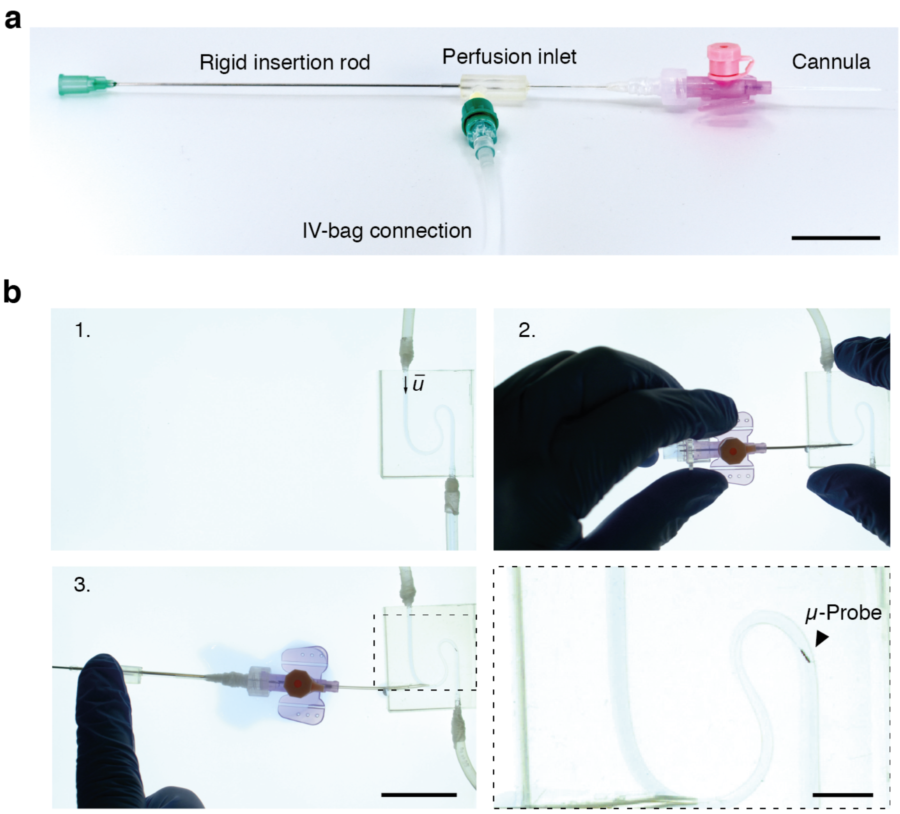

Supplementary Figure 14. **Flow-driven deployment using off-the-shelf equipment. a** A standard cannula was connected to the insertion device that is perfused by a standard pressurized IV-bag. **b** Snapshots of a cannulation process of a PDMS phantom. Scale bars, 30 mm and 5 mm (inset).

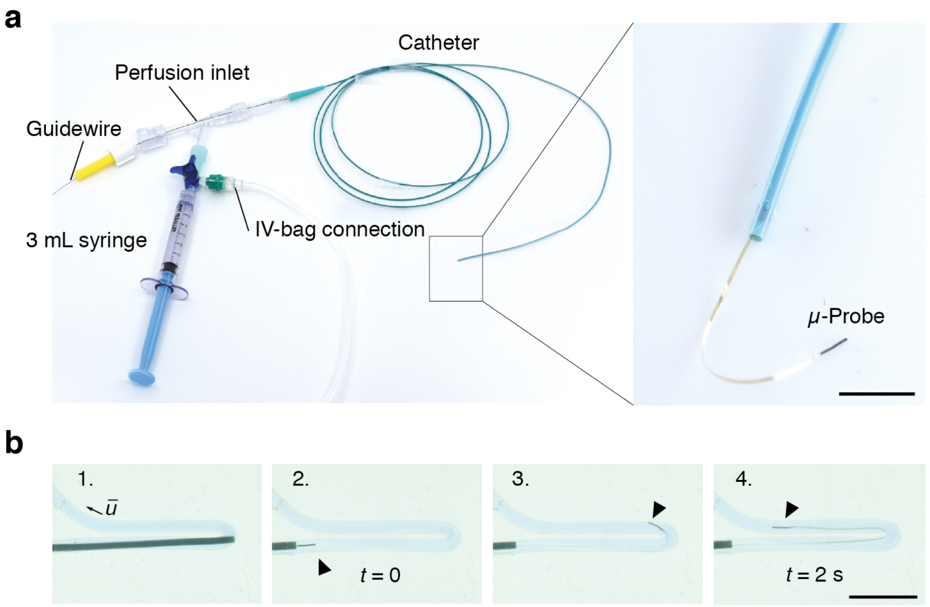

Supplementary Figure 15. **Flow-driven deployment using commercially available endovascular catheter. a** A 3 F catheter is connected to a perfusion syringe and a pressurized IV-bag. The μ-probe (Ø150 μm x 2 mm magnetic head) is glued to the guidewire and pulled into the catheter. Upon introduction of perfusion, pushing the guidewire allows the μ-probe to exit the catheter (right). **b** Demonstration of the hybrid catheter/μ-probe device. Once the catheter reach places that it cannot advance (*e.g.* a high curvature turn), it can deploy the μ-probe that can effortlessly advance with the flow and reach the target location. Scale bars, 5 mm.

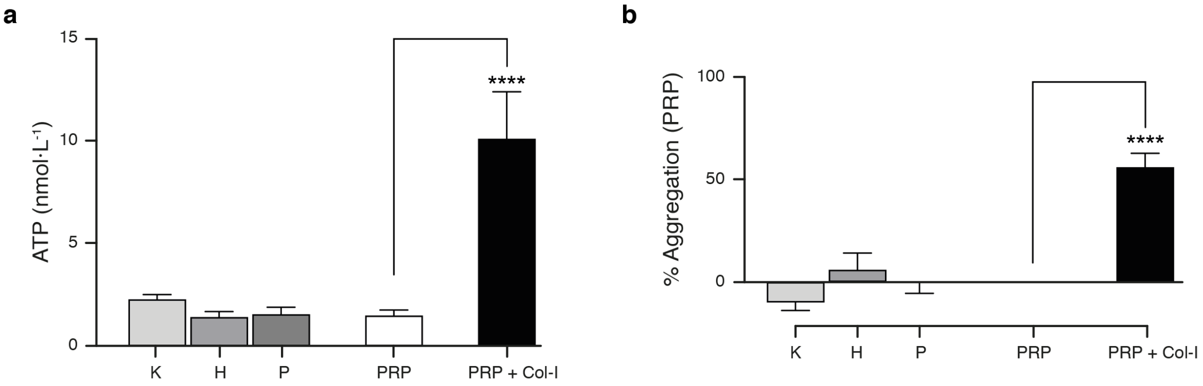

Supplementary Figure 16. **Blood compatibility assay performed on fresh human blood under high shear stress condition.** Three different samples were tested, kapton film (K: 0.004 x 0.2 x 10 mm^3), magnetic head with silica-coated magnetic particles (H: ∅0.3 mm x 1 mm), and whole μ-probe (P: K + H). Negative and positive control are platelet rich plasma (PRP – non-activated platelets) and PRP exposed to collagen type-I (PRP+Col-I – activated platelets). **a** ATP release from platelets was measured using luciferin-luciferase reaction. **b** Platelet aggregation was measured using absorbance (595 nm). The aggregation % was defined by the transmission of light through non-activated PRP, represented as 0 % aggregation, and through platelet-poor plasma (PPP) as 100 % aggregation. Both techniques have been vastly used in the platelet field to investigate the thrombogenicity of different materials and devices. Values were compared with the non-activated platelet, (***P < 0.001, PRP) by one-way ANOVA followed by Dunnett's post hoc test (SEM, duplicate average from four independent experiments).

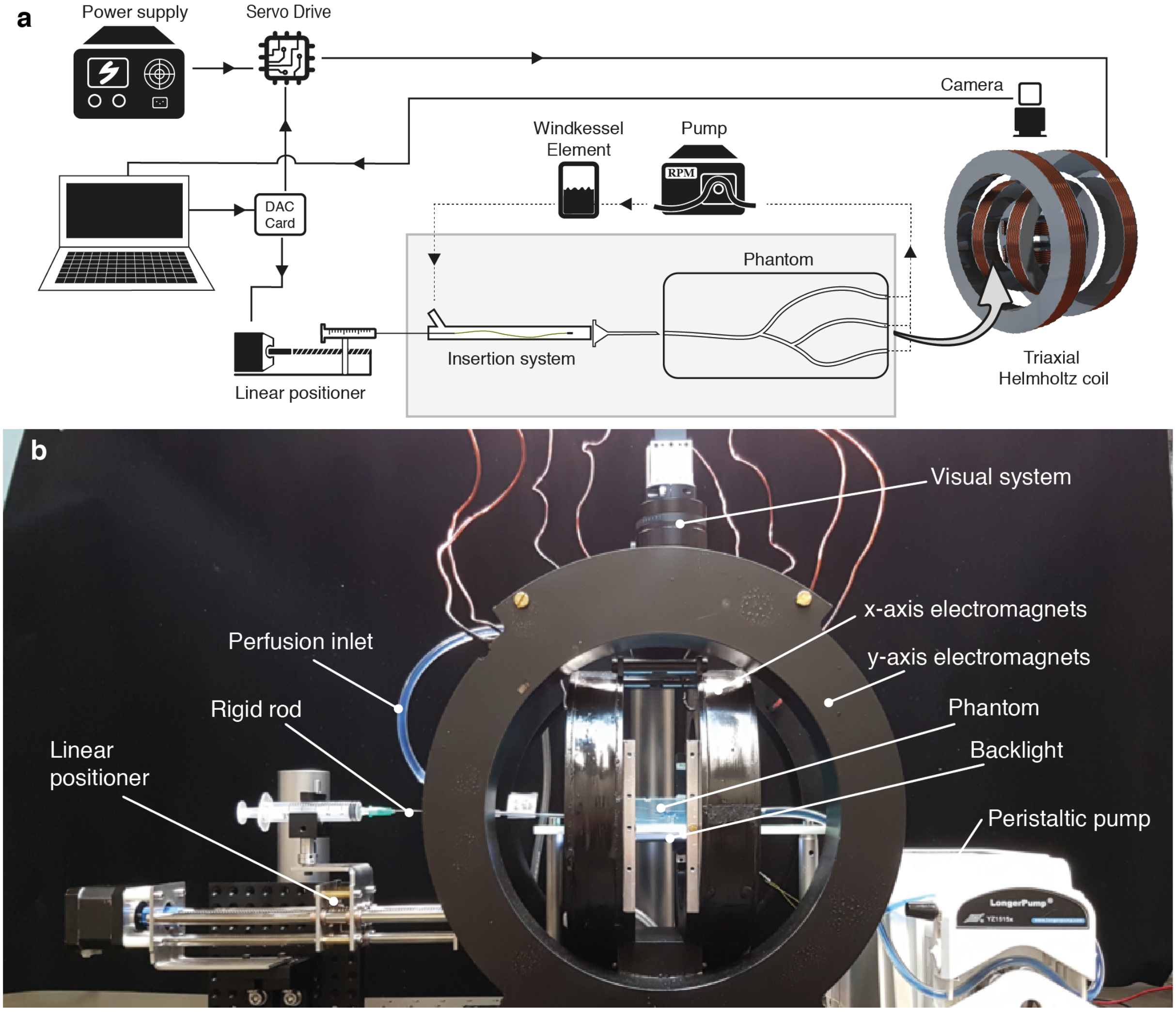

Supplementary Figure 17. Experimental platform. **a** Digital to Analog Converter (DAC) card allows real-time communication between the computer and the hardware. The servo drives adjust the current provided by the power supplies to the coils. The linear positioner allows controllable release of the μ-probe through the rigid rod of the insertion system. Windkessel system is placed between the peristaltic pump and the perfusion-based insertion system to dampen the flow oscillations from the pump. Visual feedback for teleoperation is provided by a CMOS camera. Drawings are not to scale. **b** Picture of the experimental platform.

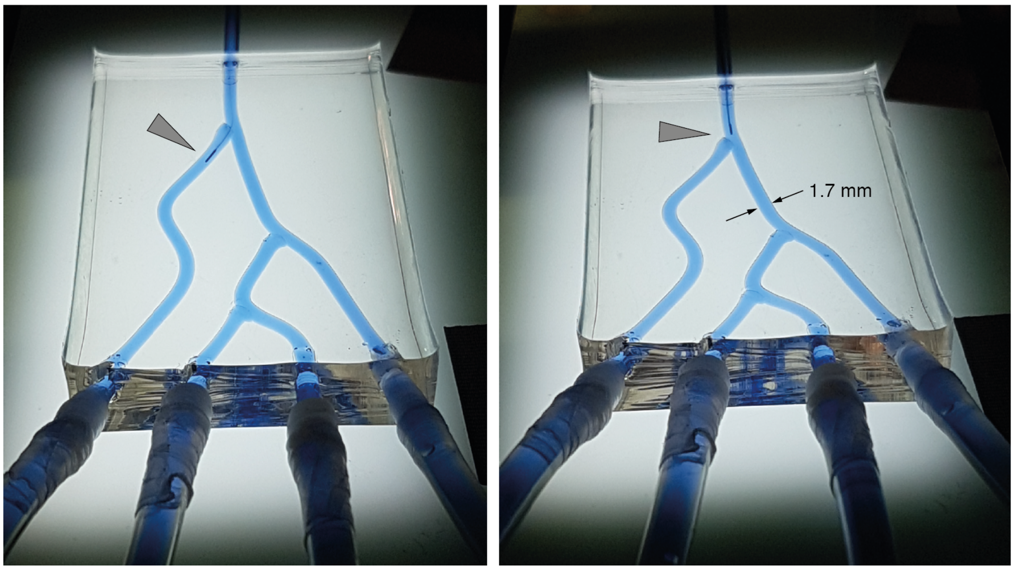

Supplementary Figure 18. Illumination system for biomimetic phantoms. Backlight system provides optimal contrast for visualizing the μ-probe inside transparent channels. Grey arrows indicate the position of the μ-probe head.

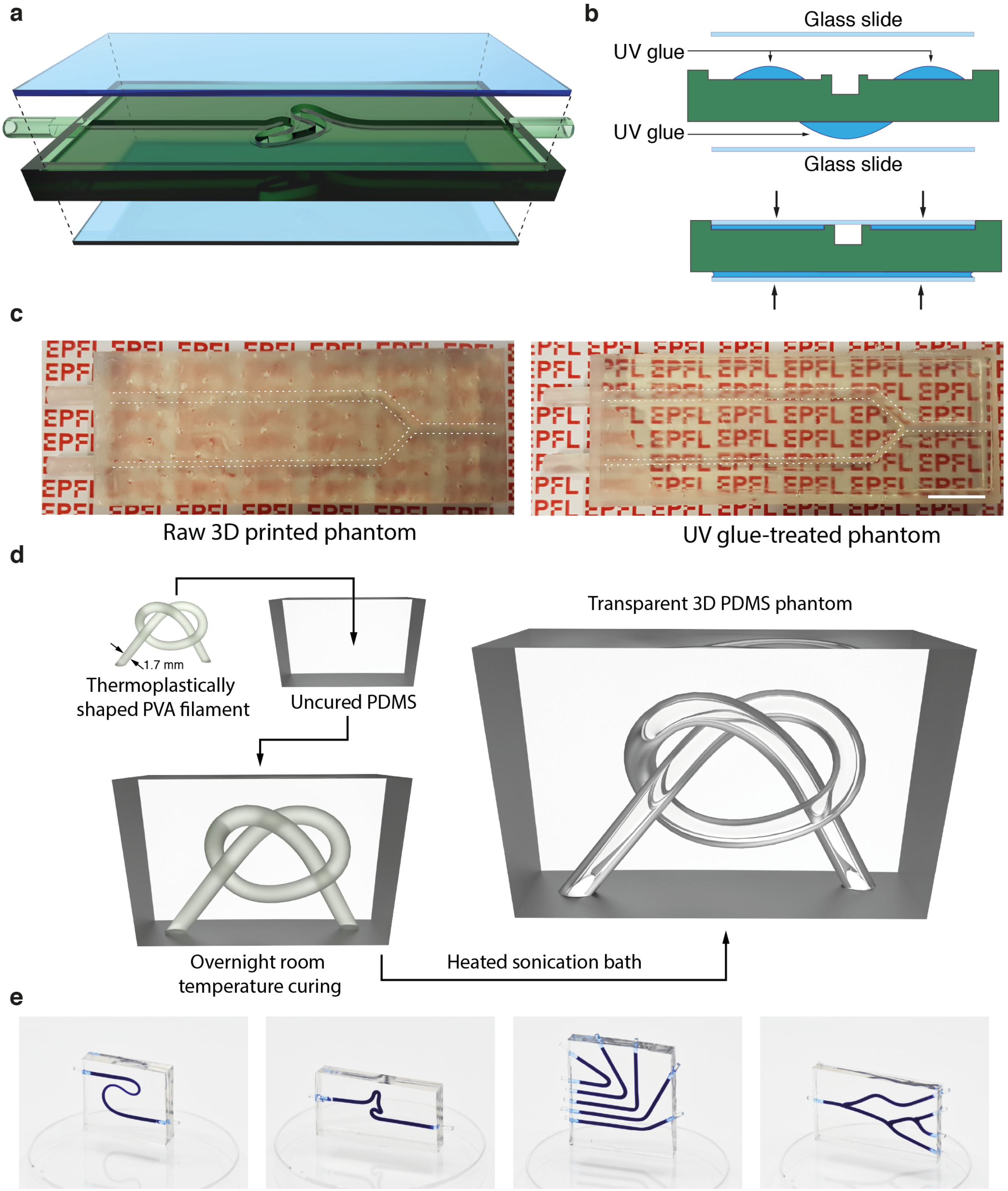

Supplementary Figure 19. Fabrication of biomimetic phantoms. The 3D printed open channel is sandwiched between two glass slides and laminated using UV glue as shown in the (**a**) oblique and (**b**) side views. **c** Refractive index-matching UV glue provides optical transparency for visualization. Difference in transparency of a 3D printed phantom prior (left) and after (right) the addition of UV glue is shown. Scale bar, 1 cm **d** Process flow for manufacturing the elastomer phantoms. Commercially available 1.75 mm-diameter polyvinyl alcohol (PVA) filament is thermoplastically shaped and bonded. Sonication in a heated bath dissolves the PVA inside the casted elastomer, which was removed with extensive washing. **e** Pictures of representative devices. Channels are made visible with a blue ink.

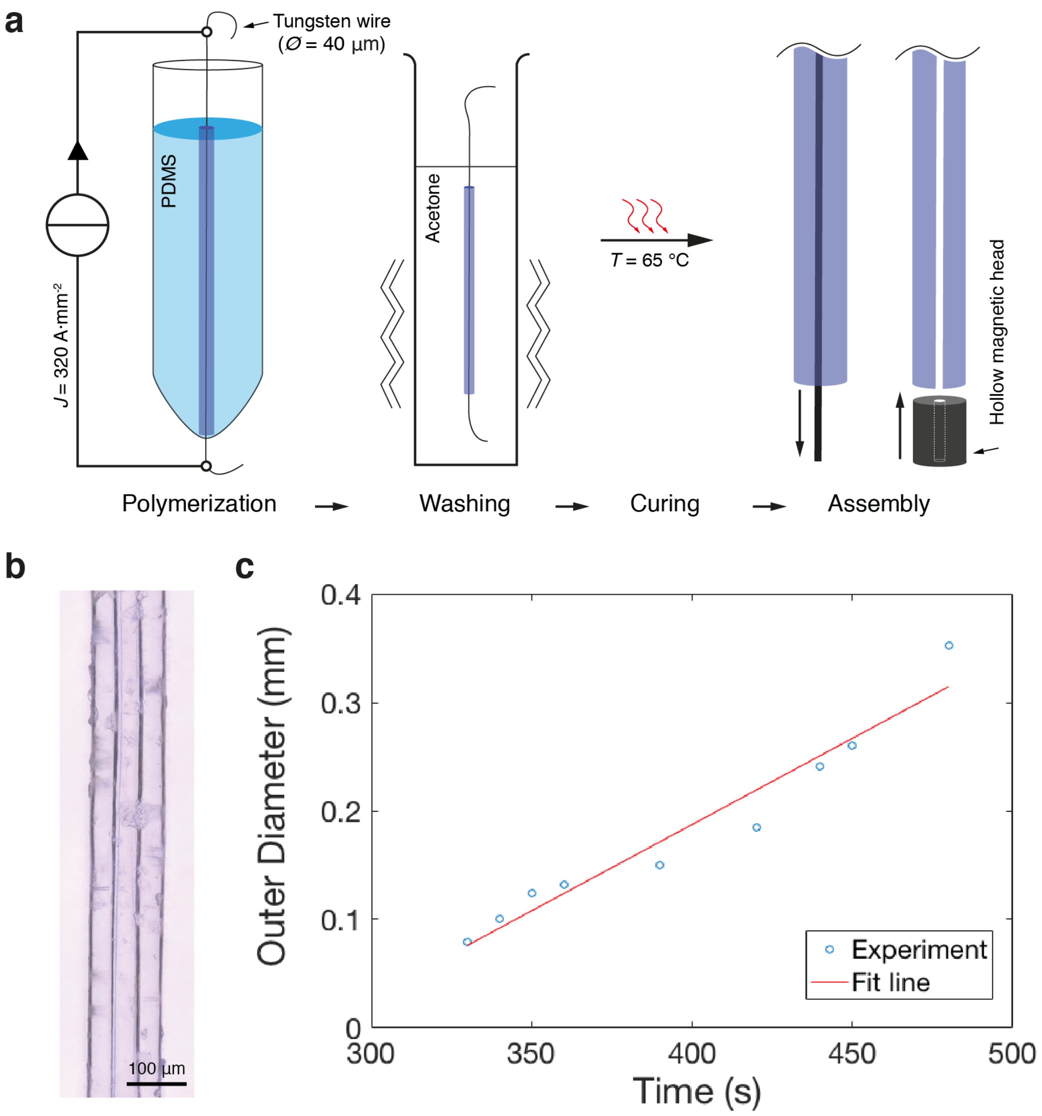

Supplementary Figure 20. Fabrication of μ-catheters a Elastomer tubes are fabricated through controlled Joule heating of a tungsten wire. The magnetic tubular head is glued to the tube. Drawings are not to scale. **b** Close-up picture of a microfluidic μ-catheter with an inner diameter of 40 μm and outer diameter of 120 μm. Scale bar, 100 μm. **c** Calibration curve between the obtained outer diameter of the μ-catheter with respect to the curing time at a current density of 320 A·mm^{-2} through a 40 μm-diameter tungsten wire.

Supplementary Tables

Supplementary Table 1. Flow velocity and shear stress measurements. Comparison between the analytical and empirical values of flow velocity and wall shear stress under different conditions. Flow velocity and shear stress were calculated from the input flow velocity and measured using the microfabricated flow sensor. Number in parenthesis corresponds to the incremental factor with respect to the levels in the pre-stenosis or vessel 1.

	Dimensions (mm x mm)	Average velocity $\bar{u}$ (cm·s^{-1})			Wall shear stress τ_{wall} (Pa)			
	w · h	**Calculated**	**Measured**	***Error***	**Calculated**	**Measured**	***Error***	
**Pre-stenosis**	2 x 2	4.3	4.2	2.3%	0.191	0.187	2.1%	**Stenosis**
**Stenosis**	1 x 1	17.3 (4x)	29.8 (7x)	72.3%	1.530 (8x)	1.317 (7x)	13.9%	**Stenosis**
**Vessel 1**	2 x 2	20.4	18.9	7.4%	0.901	0.860	4.6%	**Bifurcation**
**Vessel 2**	2 x 2	16.7 (0.8x)	15.0 (0.8x)	10.2%	0.738 (0.8x)	0.675 (0.8x)	8.5%	**Bifurcation**
**Vessel 3**	1 x 1	17.3 (0.8x)	31.2 (1.7x)	80.3%	1.526 (1.7x)	1.388 (x1.6)	9.0%	**Bifurcation**

Supplementary Notes

Supplementary Note 1. Computational modelling of µ-probe dynamics.

In our platform, the filament dynamics is driven by the interplay of four physical effects - elastic bending forces, contact forces, magnetic torques, and viscous stresses. We assume external forces, such as gravity, to be negligible with respect to viscous forces. The primary output is the stationary position of a flexible fiber in a two-dimensional channel with viscous flow. The solid was modeled as a 2D beam under pure bending. Local curvatures of the deformed beam were computed using the moment-curvature relationship, and the full geometry was solved in a geometrically non-linear frame. The length L of the µ-probe is assumed to be much larger than the transverse dimensions. The filament is considered to be inextensible and positioned in a two-dimensional flow of velocity $\mathbf{U}$, dynamic viscosity μ and density ρ. The flow velocity at each point in the channels was computed by solving the incompressible steady-state Navier-Stokes equations using computational fluid dynamics. 2D CFD simulations were performed using an open source software (OpenFOAM). The stationary pose of the µ-probe in the channel was obtained by iteratively solving the beam mechanics while probing the fluid velocities at each node of the structure at each iteration. The problem was considered to be quasi-static (this hypothesis was validated with the experimental data), and the time-dependent solution is simple obtained by concatenating a series of stationary states. This approach allows to obtain time-dependent solutions in a fast an efficient manner by neglecting dynamic effects. We implemented a one-way fluid-structure interaction in the simulations, *i.e.* the µ-probe deformation and motion have no impact on the fluid flow.

The filament is discretized and the fluid velocity at each node is known and can be projected onto a local coordinate system (see Fig. 3a). It is then possible to obtain the local velocities at the node i from the velocity vector $\mathbf{U}$: $U_{\perp}^{i}$ and $U_{\parallel}^{i}$. The forces in this local coordinate system become:

$$F_{\perp}^{i} = \frac{1}{2} \cdot \mathrm{h}^{i} \cdot \rho \cdot \xi_{\perp} \cdot l^{i} \cdot |U_{\perp}^{i}| \cdot U_{\perp}^{i} \tag{1}$$

$$F_{\parallel}^{i} = \frac{1}{2} \cdot \mathrm{h}^{i} \cdot \rho \cdot \xi_{\parallel} \cdot l^{i} \cdot |U_{\parallel}^{i}| \cdot U_{\parallel}^{i} \tag{2}$$

where *h* is one of the body lengths (2*R* for the cylinder and a for the ribbon). Using the local Reynolds numbers $\mathrm{Re}_{\perp} = \frac{\mathrm{h}^{i} \cdot \rho \cdot |U_{\perp}^{i}|}{\mu}$ and $\mathrm{Re}_{\parallel} = \frac{\mathrm{h}^{i} \cdot \rho \cdot |U_{\parallel}^{i}|}{\mu}$, the drag coefficients $\xi_{\perp}$ and $\xi_{\parallel}$are calculated as[1]:

$$\xi_{\perp} = \begin{cases} \frac{8\pi}{s}\left(1 - \frac{s^2 - \frac{s}{2} + \frac{5}{16}}{32s}\right)\mathrm{Re}_{\perp}{}^{2}, & 0.01 < \mathrm{Re}_{\perp} < 1 \\ 2\exp\left(\sum_{j=0}^{3} p_{a,j} (\ln \mathrm{Re}_{\perp})^{j}\right), & 1 < \mathrm{Re}_{\perp} < 20 \\ \frac{4}{\sqrt{\mathrm{Re}_{\perp}}} + 1, & \mathrm{Re}_{\perp} > 20 \end{cases} \tag{3}$$

$$s = 2.002 - \ln(\mathrm{Re}_{\perp}) \tag{4}$$

$$\xi_{\parallel} = \begin{cases} \frac{8\pi}{2s-1}\left(1 - \frac{2s^2 - 2s + 1}{16(2s-1)}\right)\mathrm{Re}_{\parallel}{}^{2}, & 0.01 < \mathrm{Re}_{\parallel} < 1 \\ 2\exp\left(\sum_{j=0}^{3} p_{b,j} (\ln \mathrm{Re}_{\parallel})^{j}\right), & 1 < \mathrm{Re}_{\parallel} < 20 \\ \frac{5.4}{\sqrt{Re_{\parallel}}}, & Re_{\parallel} > 20 \end{cases} \tag{5}$$

$$s = 2.002 - \ln(\mathrm{Re}_{\parallel}) \tag{6}$$

$p_{a,0}$	1.6911
$p_{a,1}$	$-6722 \cdot 10^{-1}$
$p_{a,2}$	$3.3287 \cdot 10^{-2}$
$p_{a,3}$	$3.5015 \cdot 10^{-3}$

$p_{b,0}$	1.1552
$p_{b,1}$	$-6.8479 \cdot 10^{-1}$
$p_{b,2}$	$1.4884 \cdot 10^{-1}$
$p_{b,3}$	$7.4966 \cdot 10^{-1}$

Supplementary Table 2 Parameters used to determine the drag coefficients $\xi_{\parallel}$ and $\xi_{\perp}$.

The drag coefficients $\xi_{\perp}$ and $\xi_{\parallel}$ are valid for cylindrical slender bodies. However, at relatively low Re, these coefficients are expected to be similar for ribbon shaped slender bodies. Values of $F_{\perp}$ and $F_{\parallel}$ for different filaments and different flow rates were computed with the resistive force theory presented elsewhere[1]. The forces in global coordinates are given by:

$$F_x^i = \cos(\theta_{i-1}) \cdot F_{\parallel}^i - \sin(\theta_{i-1}) \cdot F_{\perp}^i \tag{7}$$

$$F_y^i = \sin(\theta_{i-1}) \cdot F_{\parallel}^i + \cos(\theta_{i-1}) \cdot F_{\perp}^i \tag{8}$$

The calculated forces were compared with the force values obtained using CFD. Filaments where modelled in COMSOL Multiphysics using the laminar flow module. The flow was considered planar in a (*X*, *Y*) plane purely along the *X* direction. The angle theta was considered to be 0° when perpendicular to the flow. These results were used to perform a weighted fit (equal weight for each node) of the $\xi_\perp$ and $\xi_\parallel$ coefficients:

$$\boldsymbol{\xi} = \lambda_\perp \cdot \xi_\perp \cdot \mathbf{e}_\perp + \lambda_\parallel \cdot \xi_\parallel \cdot \mathbf{e}_\parallel \quad (9)$$

	**Cylinders**	**Ribbons**
$\lambda_\perp$	0.95	0.95
$\lambda_\parallel$	0.65	0.63

Supplementary Table 3 Fitting parameters used for calculating the drag coefficient $\boldsymbol{\xi}$.

The amplitude of the parameters λ shows that the default model is capable of accurately estimating the normal fluid forces but tends to overestimate tangential fluid forces. By using these fitting parameters, we developed a model that can accurately calculate the fluid forces. The comparison between the CFD results and the fitted model along *X* and *Y* direction are shown in Supplementary Figures 21 and 22, respectively.

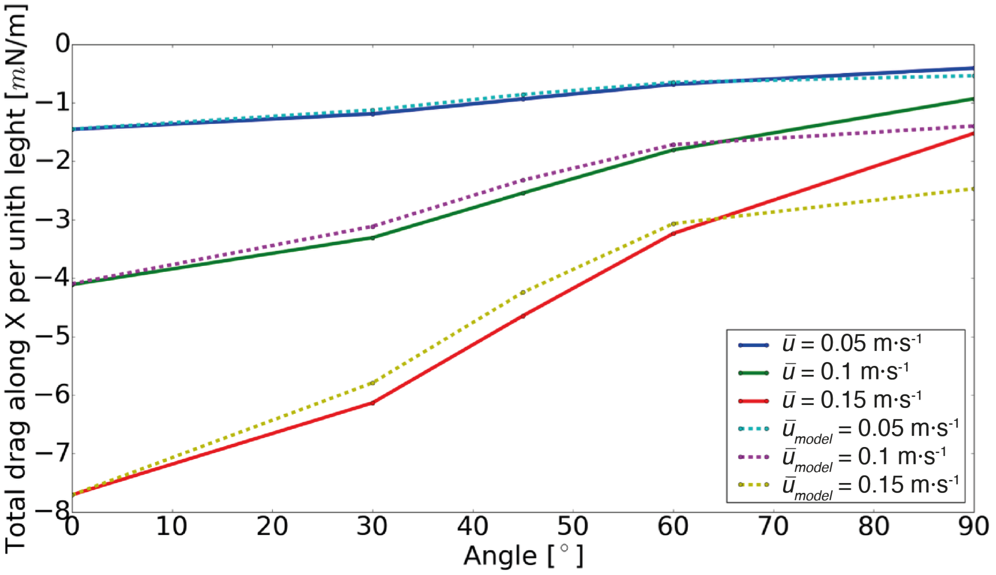

Supplementary Figure 21. The comparison between the CFD results and the fitted model along X direction. Drag per unit length along the *X* direction for cylindrical fibres of diameter of 0.25 mm for multiple fibre angles and flow velocities. COMSOL simulations and the calculation obtained with our model are shown in solid lines dashed lines, respectively.

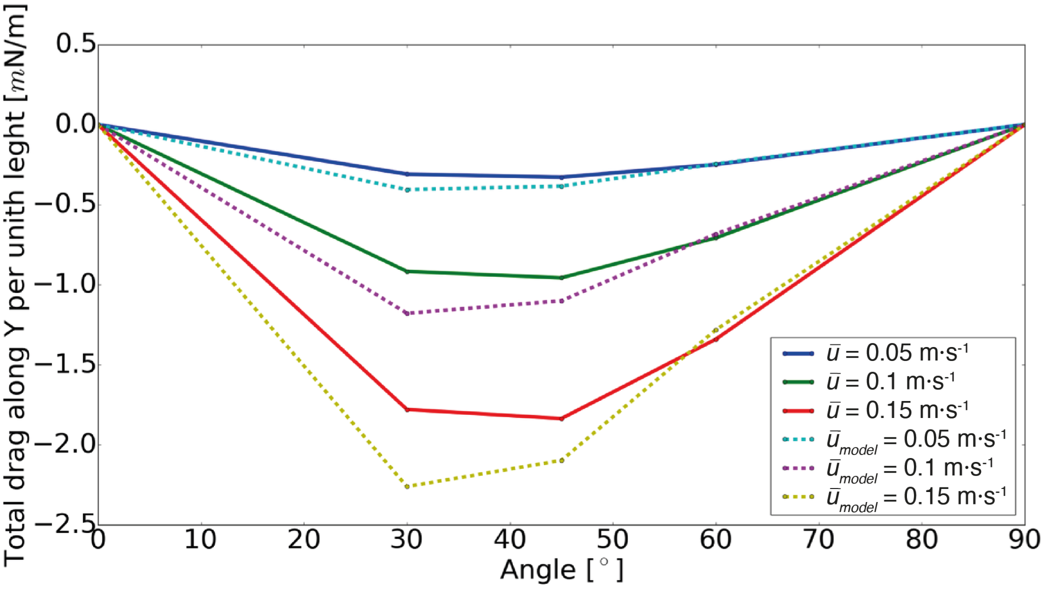

Supplementary Figure 22. The comparison between the CFD results and the fitted model along Y direction. Drag per unit length along the *Y* direction for cylindrical fibres of diameter of 0.25 mm for multiple fibre angles and flow velocities. COMSOL simulations and the calculation obtained with our model are shown in solid lines dashed lines, respectively.

The proximal tip of the μ-probe was considered to be clamped and the distal tip of the μ-probe was considered as free. With these hypotheses, it is possible to model the two-dimensional μ-probe using a geometrically non-linear approach to the pure bending theory of beams. The

local strain is considered to be small and thus linear elasticity can be considered for the beam. Shear stresses for slender beams can be ignored. The constitutive equation of the Euler-Bernoulli beam in curvilinear coordinates is $\frac{1}{\rho} = \frac{M}{EI}$, where the curvature $\frac{1}{\rho} = \frac{d\theta}{ds}$, consequently, the relationship between bending moment and beam deformation is:

$$EI\frac{d\theta}{ds} = M(s) \tag{10}$$

A lumped model derived from the Euler-Bernouilli equation can be obtained by discretizing the beam in n elements of lengths l_i with nodes with torsional stiffnesses $k_i = \frac{EI}{l_i}$. At each node the bending angle can be determined:

$$\alpha = \frac{T(M,F_x,F_y)}{k} \tag{11}$$

Where θ_i of each node directly depends on the torque T_i at that node. T_i depends on the contributions of the moments M, the horizontal forces F_x and the vertical forces F_y acting on the beam. The moment in each node is distributed to the nodes closer to the pinned end:

$$T^m_{0,1,2,\ldots,i-1} = M_i \tag{12}$$

Forces have to be transformed in torques, for $0 \leq j \leq i - 1$ the forces at each node are:

$$T_j^y = (x_i - x_j) \cdot F_{y,i} \tag{13}$$

$$T_j^x = (y_i - y_j) \cdot F_{x,i} \tag{14}$$

Finally, the relative angle at each node becomes:

$$\alpha_i = \frac{T_i^m + T_i^x + T_i^y}{k_i} \tag{15}$$

For small deformations, it is possible to analytically solve the Euler-Bernoulli equation and consequently solve the lumped model in one iteration. However, for large deformations, the loading is position dependent (geometrical non-linearities). An iterative method was implemented[2] to solve the lumped model for large deformation scenario. A relaxation factor β was used:

$$T_j^r = T_{j-1}^r + \beta \cdot \frac{T_j + T_{j-1}^r}{2} \tag{16}$$

where values of the relaxation factor β were chosen between 0.8 and 0.99 depending on the convergence rate of the problem. This algorithm was validated using COMSOL Solid Mechanics on a largely deformed beam.

The magnetic moment M_i at each node of the magnetic head was obtained by estimating the magnetization of the heat. The magnetic powder used has a residual induction $B_r = 850\ \mathrm{mT}$ that was considered to be constant in magnetic fields $H \leq 50\ \mathrm{mT}$. The magnetization is then defined as ($\mu_0 = 4\pi \cdot 10^{-7}\ \mathrm{N} \cdot \mathrm{A}^{-2}$):

$$\mathbf{m} = \frac{v\mathbf{B_r}}{\mu_0} \tag{17}$$

The magnetic torque can be expressed as:

$$\mathbf{T} = \mathbf{m} \times \mathbf{H} \tag{18}$$

For a 1 mm long and 0.35 mm in diameter magnetic head the total volume is 0.0952 mm^2 of which between 5% and 20% of the volume is magnetic material. The obtained head has a magnetization between 3 $\mu A \cdot m^{-2}$ and 13 $\mu A \cdot m^{-2}$ and the maximum torque at 30 mT is between 0.09 μN·m and 0.39 μN·m.

A simple penalty algorithm was also implemented in order to account for contact between the walls of the channel and the μ-probe. A force normal to the channel is applied on each node of the μ-probe that violates the non-penetration condition. This contact force depends quadratically on the penetration distance as follows:

$$F_x^c = \gamma \cdot \delta_x^2 \cdot \mathrm{n_x} \tag{19}$$

$$F_y^c = \gamma \cdot \delta_y^2 \cdot \mathrm{n_y} \tag{20}$$

Where the multiplication factor γ, which is between $10 \cdot 10^{-9}$ and $200 \cdot 10^{-9}$ was tuned depending on the amount of reaction force needed. These reaction forces can directly be used in the lumped model as an additional set of forces acting on the system.

Supplementary Note 2. µ-Probe navigation under limited flow velocity

Accessing geometrically unfavourable daughter vessels poses a major problem for conventional endovascular devices. We showed so far that the advancement is governed by the viscous stresses, which in turn facilitate the steering into difficult bifurcations, for instance 90° diverting daughter vessels (T-shape). However, in scenarios with unfavourable hydrodynamic conditions, *i.e.* poor or absent flow, the µ-probe may experience insufficient drag force and, therefore, has difficulty to advance further. In some cases, the proximal filament may even be suctioned in the wrong daughter vessel given a prohibitive ratio $\bar{u}_1/\bar{u}_2$ as shown with red arrows (Supplementary Fig. 23a). To overcome this limitation, the magnetic head can be controllably actuated to boost the drag force or exploit the vessel walls as anchor points. In the event that $0 < \bar{u}_1/\bar{u}_2 < 0.1$, *i.e.* fluid is predominant in vessel 2 but still present in vessel 1, the magnetic head can be orientated at a specific offset with respect to the fluid direction in order to increase drag force (Supplementary Fig. 23b). The sudden increase in drag force is sufficient to regain tensile regime in the filament and to advance the µ-probe. After successfully transporting the µ-probe in the target daughter vessel using the drag-boosting protocol, the total drag force acting on the filament becomes sufficient to sustain advancement without external help.

In total absence of flow the µ-probe can advance by crawling inside the vessel. Crawling requires synchronisation between the release of the µ-probe and the rotation of the magnetic field (Supplementary Fig. 23c). The rotating magnetic head touches the vessel wall and pulls a portion of the proximal filament inside the target vessel. The viscous stresses can then charge the filament with elastic energy, which is ultimate released upon relaxation of the magnetic torque. The result is the propulsion of the magnetic head forward. This protocol can be repeated until reaching a vessel with hydrodynamic conditions that would allow unsupervised advancement.

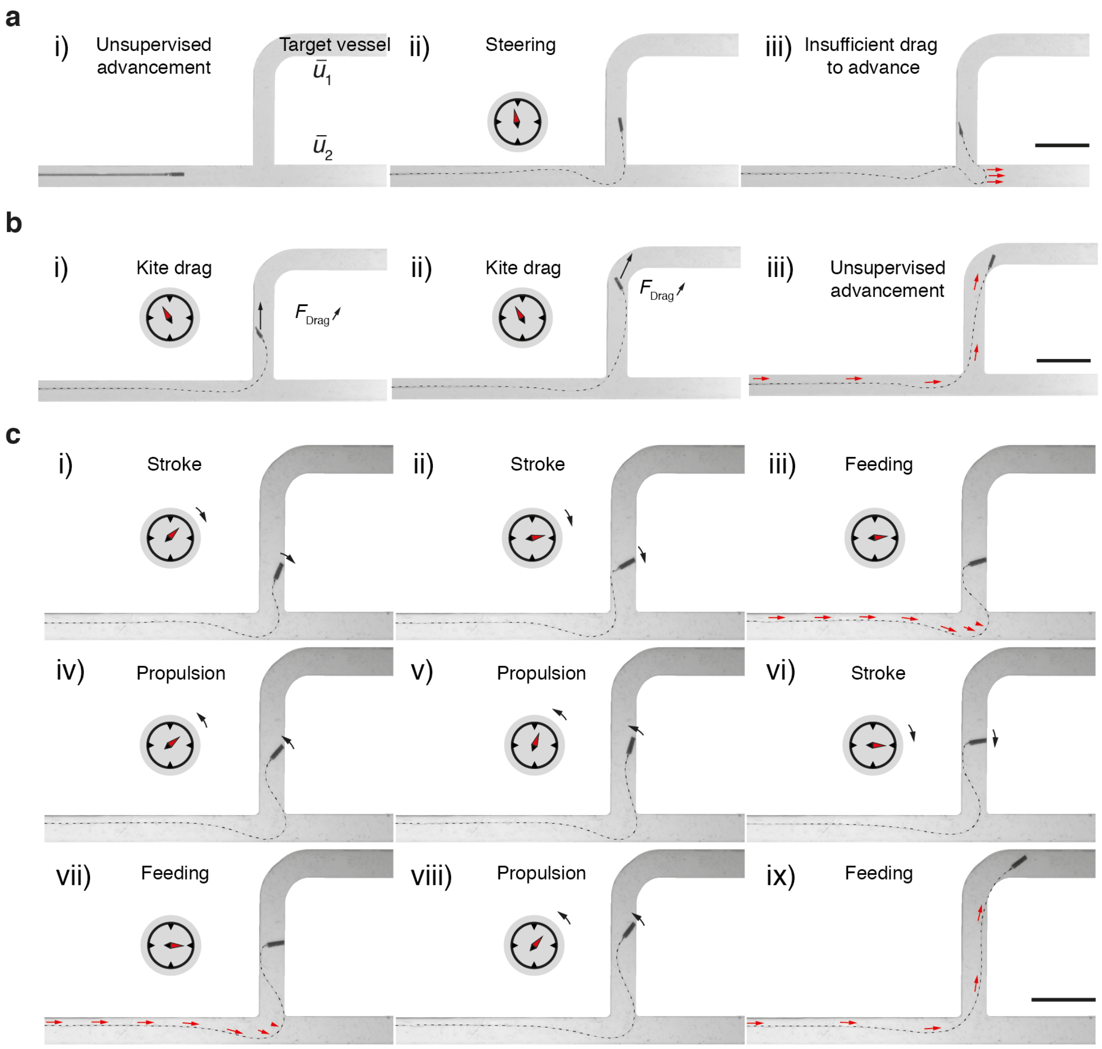

Supplementary Figure 23. Locomotion modes to advance in hydrodynamically unfavourable vessels. a The μ-probe is unable to advance into the vessel 1. **b** Kite-effect strategy to boost drag forces. **c** Crawling into the vessel 1 using magnetic actuation. Scale bars, 5 mm.

To visualize the navigation success in the T-shape bifurcation, we created a phase diagram illustrating the navigation score at different $\bar{u}_1/\bar{u}_2$ ratio and for two different head geometries channel (Supplementary Fig. 24). $\bar{u}_1$ is the average velocity in the 90°-diverting vessel and $\bar{u}_2$ the average velocity in the straight channel. We iteratively navigated the μ-probe into the target daughter artery and recorded the navigation of three different μ-probes. Each dot, dash or bar corresponds to the score result of 10 navigation runs for each μ-probe (n = 10). The results show a wide regime in which the μ-probe can efficiently navigate into the target vessel (dotted green area) regardless the total input flow. Intuitively, increasing the flow going into the target vessel facilitates the navigation. At lower $\bar{u}_1/\bar{u}_2$ ratio, and low input flow, the μ-probe can be

steered into the T-vessel but requires time-varying actuation of the head to reach the target (dashed light green area). Increasing the total input flow, completely precludes the navigation because of dominant suctioning effects transporting the proximal filament into the straight channel (crossed sky-blue area). These adverse effects became even more significant at higher input flow.

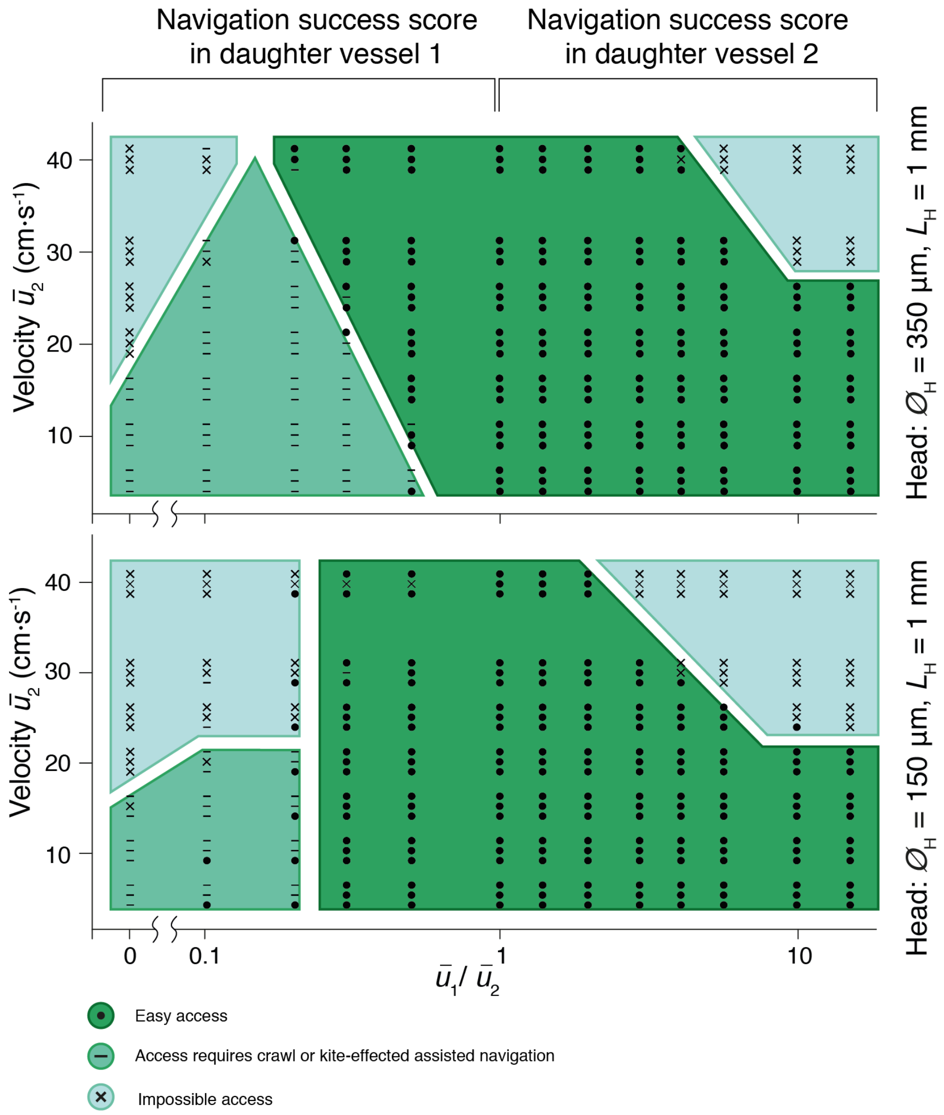

Supplementary Figure 24. Evaluation of steering success in an asymmetric T-shaped bifurcation. Phase diagram illustrating the navigation success (green area) under uneven flow distributions between the two daughter vessels. Upon hydrodynamically unfavourable conditions, the μ-probe can exploit kite effect or crawling locomotion (light green area) or be unable to continue the navigation (sky-blue area). Each symbol represents the result of 10 trials. A total of three μ-probes were tested (*n* = 30). Two different head diameters were tested: 350 μm (top) and 150 μm (bottom).

Supplementary Note 3. Flow profile in a square duct

The flow profile was calculated using the Fourier sum representing the general formula for flow velocity in rectangular ducts[3]:

$$U_x(y,z) = \frac{4h^2\Delta p}{\pi^3 \eta L}\sum_{n,odd}^{\infty}\frac{1}{n^3}\left[1-\frac{\cosh(n\pi y/h)}{\cosh(n\pi w/2h)}\right]\sin\left(\frac{n\pi z}{h}\right) \quad (21)$$

$$For \qquad -\frac{1}{2}w < y < \frac{1}{2}w \qquad and \qquad 0 < z < h \quad (22)$$

Where $\Delta p/L$ is the pressure drop along the duct, η is the dynamic viscosity of the fluid, *w* and *h* are the channel y and z dimensions respectively. Flow rate *Q* and $\bar{u}$ can be found by integration:

$$Q = \frac{\bar{u}}{wh} = \frac{h^3 w\Delta p}{12\eta L}\left[1-\sum_{n,odd}^{\infty}\frac{1}{n^5}\frac{192}{\pi^5}\left(\frac{h}{w}\right)\tanh\left(\frac{n\pi w}{2h}\right)\right] \quad (23)$$

The velocity profile is then computed by substituting Equation (23) in Equation (21). The 3D velocity profile and the corresponding 2D flow profile $U_x(y=0,z)$ are shown in Supplementary Figure 25.

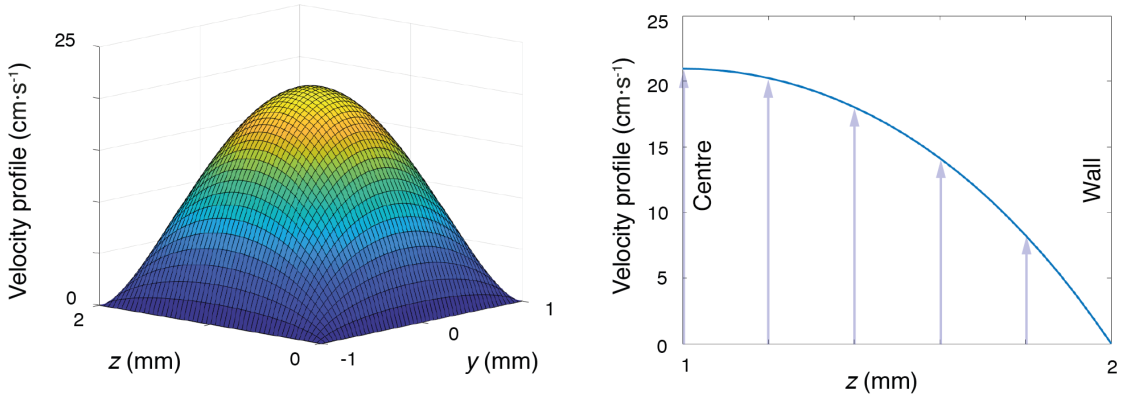

Supplementary Figure 25. Flow profile computation. 3D flow profile of inside a 2 mm x 2 mm channel (left) and its 2D centre line at *y* = 0. Input flow velocity is $\bar{u}$ = 10 cm·s^{-1}.

Finally, τ_{wall} can be extracted by interrogating the first derivative of the flow profile at the boundaries of the centreline (*z* = 2):

$$\tau_{\text{wall}} = \mu \left.\frac{\partial U_x(y=0,z)}{\partial z}\right|_{z=2} \quad (24)$$

Supplementary Note 4. Flow sensor

The working principle of the flow sensor is based on the measurement of the change in the resistance of the sensor element (due to an increase in temperature) as a response to the controlled release of heat by the heater element. The heater element is generating heat because of the current passing through a high-resistance serpentine circuit (Joule heating), which allows us to precisely control the temperature injected into the stream. Similarly, the sensor element consists of another serpentine that reports temperature changes by changing its resistance. The heater element is placed upstream with respect to the sensor and it generates a very short (~10 ms) heat pulse (Fig. 6b) that is transported downstream by the fluid flow. Higher flow rates lead to shorter travelling times, which is defined by Time-of-Flight (TOF). Figure 6b shows the time it takes for the sensor to react to the heat pulse after its generation at different flow rates.

To quantitatively verify that the thermal boundary layer was significantly smaller than the flow boundary layer, we developed a finite-element method (FEM) model of the system and analysed the temperature front around the device. Supplementary Figure 26a illustrates the position of the µ-probe inside the channel during the experimental recordings and the region that the computational model is based on. In the numerical model, the shear stress sensor is assumed to contact the vessel wall surface (y = 0). The conceptual flow profile is marked with white arrows for a 2 mm width channel and an average flow rate of $\bar{u}$ = 0.5 m·s^{-1} (Supplementary Fig. 26b). The simulation results show the interval with the largest heat front (at t = 39 ms from the heat pulse start) and highlights the highly local sensing principle of our sensor. Notably, the thermal boundary layer is considerably smaller than the flow boundary layer. To better visualize the temporal heat propagation, we zoomed in the red dotted box and showed sequential images of the fast measure. The time-lapse close-up images shown in Supplementary Figure 26c quantify the millisecond heat front propagating from the heater towards the sensor unit. At t = 39 ms and only 70 µm away from the shear stress sensor, the temperature has basically already reached the ambient temperature. After 150 ms from its release, the heat has almost completely dissipated. The input pulse generated by the heater is displayed in Supplementary Figure 26d, which is a one-to-one replicate of the input shown

in Figure 6b. The extracted TOF values are plotted with respect to the shear stress (Supplementary Fig. 26e).

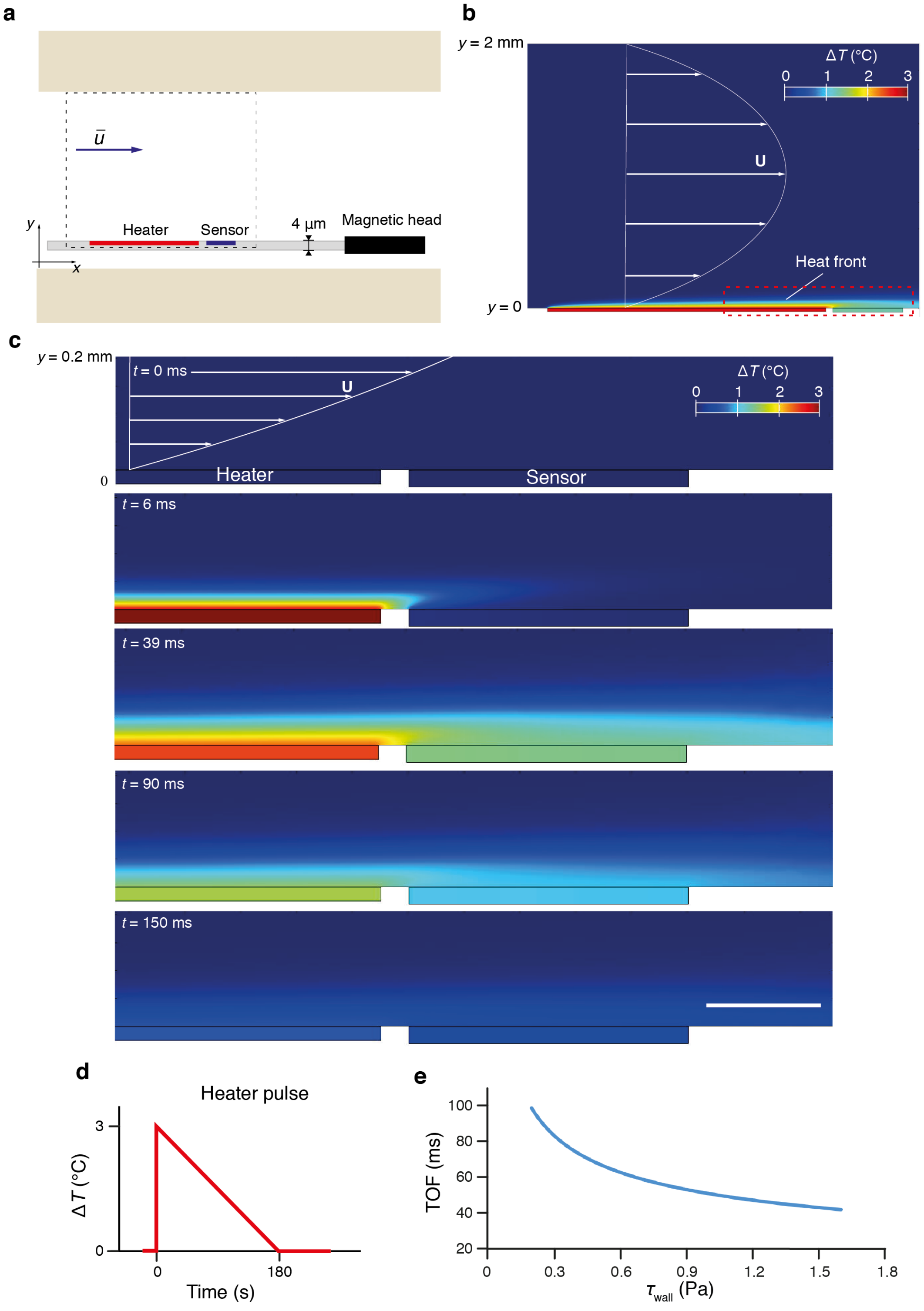

Supplementary Figure 26. **Computational analysis of the flow sensor. a** The configuration of the μ-probe inside the channel. Black dotted box denotes the part of the device and workspace that is modelled using a multiphysics simulation software. Drawings are not to scale. **b** A snapshot from the simulation showing the local heat distribution. The sensor works with low heat generation that leads to a temperature increase of 3 °C on the surface of the sensor. Heat is rapidly and locally dissipated. **c** Close-up, time-lapse images from the simulation of the area inside the red box shown in (**b**). Scale bar,

200 µm. **d** Pulse generated by the heater during the simulations. The corresponding TOF calibration curve is shown in (**e**).

Supplementary Note 5. Force analysis

There are three different situations where the µ-probe may cause damage to the vessel:

Advancement (Supplementary Note 5.1). The flow pulls or pushes the µ-probe against the vessel and causes perforation. This may potentially happen at bifurcations or corners with high curvature.

Retraction (Supplementary Note 5.2). While retracting the µ-probe against the flow, friction forces may induce dissection on the inward wall of the turns.

Magnetic Steering (Supplementary Note 5.3). Magnetic torque that is accidentally applied in the wrong direction pushes the magnetic head against the wall and causes perforation.

Supplementary Note 5.1. During advancement, the µ-probe is transported by the hydrodynamic drag force, F_{D}, which applies a distributed tension (pull force) along the entire structure. The normal forces F_{N} at the corners where the µ-probe contacts the wall scales with the relative velocity Δu of the fluid with respect to the µ-probe

$$F_{\mathrm{N}} \propto F_{\mathrm{D}} \propto (\Delta \bar{u})^{k} = (\bar{u}_{\mathrm{fluid}} - u_{\mathrm{advancement}})^{k} \tag{25}$$

where $\bar{u}_{\mathrm{fluid}}$ is the velocity of the fluid and $u_{\mathrm{advancement}}$ is the rate at which the µ-probe is released. The value of k depends on the Reynolds number as described in Equations (3) and (5). The tension on the µ-probe and the normal forces on the inward wall of the turns is maximized when $u_{\mathrm{advancement}} = 0$ and $\bar{u}_{\mathrm{fluid}}$ is larger than 0. Here, we assume $u_{\mathrm{advancement}} = 0$ to take into consideration the highest normal forces that may act on the µ-probe.

To calculate the normal forces acting on the vessel walls, we had to quantify the drag forces as a function of µ-probe length. To this end, we developed an experimental platform that consists of a straight channel where the µ-probe was inserted and exposed to flow with constant velocity (Supplementary Fig. 27a). The µ-probe was clamped to an external force

sensor (SI-KG7, WPI) that is sensitive enough to report the sub-mN tension on the µ-probe, which is solely determined by the fluid forces as the µ-probe does not contact the channel walls. The plot shown in Supplementary Figure 27b reports the total drag force per µ-probe length for the following parameters: $\bar{u}$ = 10 cm·s^{-1}, µ-probe width h = 200 µm, dynamic viscosity μ = 1 mPa·s, density ρ = 1000 kg·m^{-3}. We provided an analytical model in the Supporting Information (Supplementary Note 1) that is based on resistive force theory. Assuming Re larger than 20, the drag force can be calculated using the following formula:

$$F = 2.7 * (h\rho\mu)^{0.5} * U^{1.5} * l \quad (26)$$

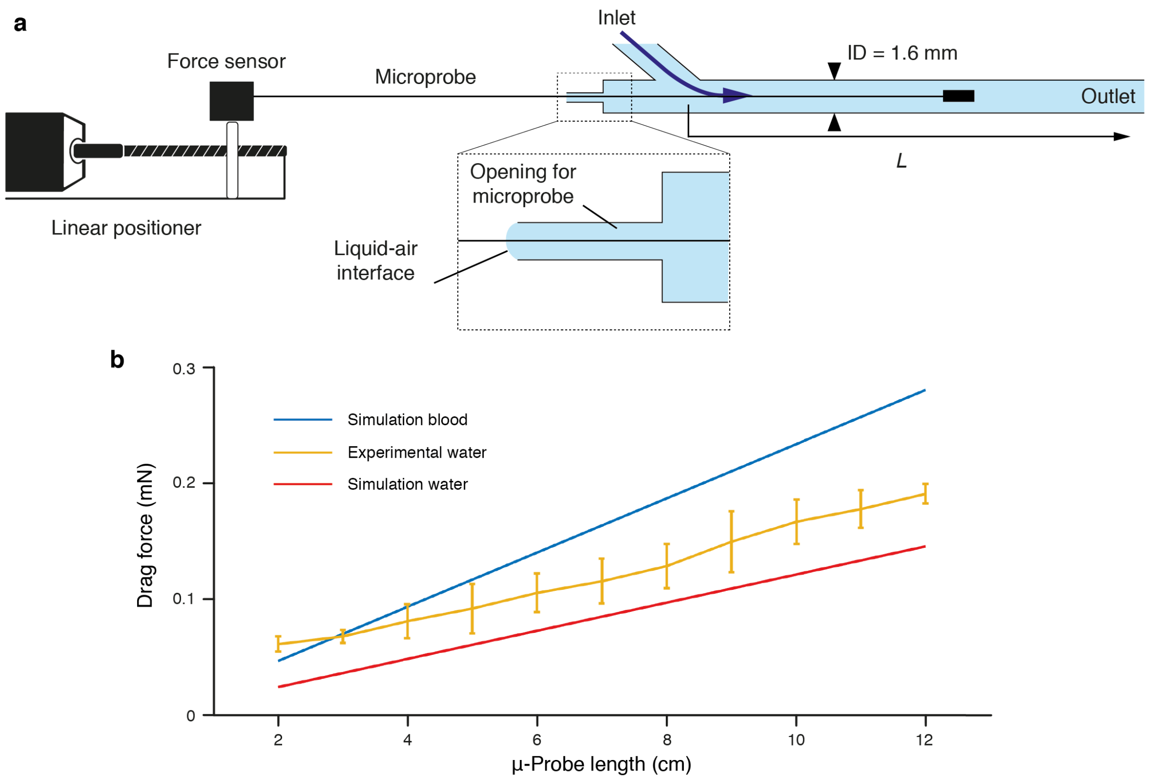

Supplementary Figure 27. **Quantification of the drag force**. **a** Illustration showing the experimental setup with the force sensor, advancement mechanism, and the fluidic device. Drawings are not to scale. **b** Experimental data and simulation of the total drag force acting on a µ-probe subjected to an average flow rate of $\bar{u}$ = 0.1 m·s^{-1} at Re > 20. The µ-probe length is the part of the µ-probe subjected to viscous stresses. Each experimental measurement was repeated at least three times.

The experimental results are in good agreement with the calculated values and the observed offset between simulations and experimental data (on average 30 µN) is due to the fact that we ignored the magnetic head in our calculations. After validating the analytical model, we calculated the drag force exerted on a 12 cm-long µ-probe by the blood flow (μ = 3.5 mPa·s,

$\bar{u}$ = 10 cm·s^{-1}, density ρ = 1060 kg·m^{-3}). The total drag force is $F_{\mathrm{N}} = 0.28$ mN, which is orders of magnitudes lower that push forces involved in the advancement of conventional guidewires[4,5]. In addition, we assumed that all the forces applied by the fluid will be transmitted to the vessel wall, which is very unlikely due to mechanical instabilities.

We next studied the normal forces generated by the μ-probe that is being pulled by the flow on the inward wall of a turn. In this configuration, the highest normal forces are recorded in a single turn channel. As an extreme example, in a 180° turn, the total force acting on the inward wall is equal to the total drag force acting on the part of the μ-probe distal to the turn. In general, the normal force can be written as $F_{\mathrm{N}} = F_{\mathrm{D}} \sin\frac{\alpha}{2}$, where α corresponds to the turn angle. The total normal force for α = 180° (see Supplementary Fig. 28) is calculated as 0.66 mN with a L = 10 cm-long μ-probe in a vessel filled with blood and under a velocity of 20 cm·s^{-1}. The forces induced on the wall during advancement are again orders of magnitude smaller than forces reported in standard catheterization techniques[4,5].

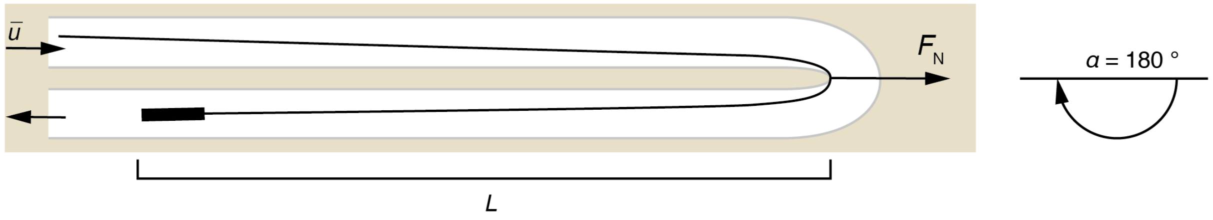

Supplementary Figure 28. **Schematic representation of a scenario where highest forces are expected**. Normal forces generated at a 180 ° sharp turn due to the drag force acting on the μ-probe of length L. Drawings are not to scale.

Supplementary Note 5.2. During retraction, a pulling (retraction) force must be exerted at the proximal clamped end, which applies friction and normal forces to the vessel at the contact points. The blood flow counteracts the retraction forces, and therefore, increases the amount of force required to pull the μ-probe compared to the case without the flow. We developed a model based on force-balance equations to study the total retraction forces required to pull back the μ-probe. We compared the calculations with the measured values of retraction forces and validated the model.

The retraction forces can be estimated from the Capstan equation, by assuming the inward wall of the turns as a series of cylinders around which the filament is wound. The angle and curvature of the turn dictates the arc surface where the μ-probe induces pressure, hence friction. See Supplementary Figure 29 for an illustration.

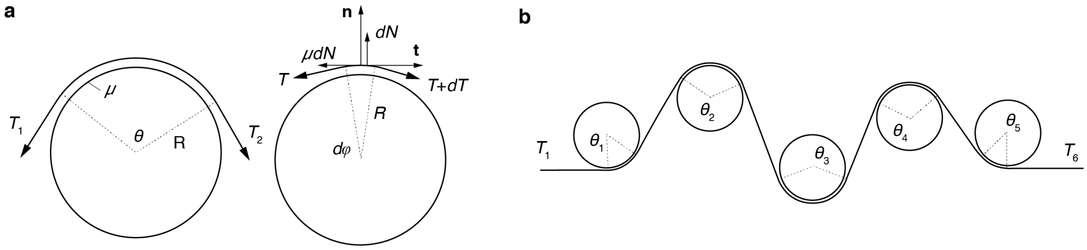

Supplementary Figure 29. **Calculation of tension on a fixed capstan system**. **a** Filament is assumed to slide on a circular object of radius *R* forming a contact arc region with angle θ (left). The dynamic friction coefficient μ decreases the tension forces across the turn from T_2 to T_1. Infinitesimal representation of the filament with $d\varphi$ as the contact angle, *dN* the normal force, μdN the dynamic friction and *dT* as the difference between the tension forces across the turn (right). The reference frame is the normal ***n*** and the tangential ***t*** directions. **b** Series of turns with contact angle θ_i between the filament and the object.

The force balance along the tangential (***t***) and normal (***n***) axis leads to

$$d\mathrm{F_N} = T\,d\varphi \tag{27}$$

$$dT = \mu\,d\mathrm{F_N} \tag{28}$$

where F_N is the normal force, *T* is the tension on the rope, μ is the dynamic friction, and θ is the total angle between the inward wall of the artery and the μ-probe. Equations (27) and (28) can be combined as:

$$\int_{T_1}^{T_2} \frac{dT}{T} = \exp\left(\mu\theta\right) \tag{29}$$

The solution of the integral leads to the Euler-Eytelwein formula[6] or Capstan equation, that expresses the tension drop across each turn:

$$\frac{T_2}{T_1} = \exp\left(\mu\theta\right) \tag{30}$$

For a series of *n* turns, the cumulative tension T_{Tot} (*i.e.* force required to retract the μ-probe) is given by:

$$F_{\mathrm{R}} = T_{\mathrm{Tot}} = \; T_1\, e^{\mu \sum \theta} \; = \; T_1\, e^{\mu(\theta_1 + \,\theta_2 + \cdots + \,\theta_n)} \tag{31}$$

In our experimental setup, the tension T_1 corresponds to the drag force acting on the μ-probe before entering the first turn, $F_{\mathrm{D,Baseline}}$. Thus,

$$F_{\mathrm{R}} = F_{\mathrm{D,Baseline}}\ e^{\mu \sum \theta} = F_{\mathrm{D,Baseline}}\ e^{\mu(\theta_1 + \theta_2 + \cdots + \theta_n)} \tag{32}$$

As described above, the highest normal forces are recorded in the first turn of the channels. The total normal force exerted to the wall on the first turn is calculated by integrating the total pressure (*p*) acting on the contact arc:

$$F_{\mathrm{N}} = R \int_0^{\varphi} p(\beta) d\beta = \frac{T_1}{\mu}\left[e^{\mu\theta} - 1\right] = \frac{F_R}{\mu}\left[e^{\mu\theta} - 1\right] \tag{33}$$

To validate the model, we devised a setup where the μ-probe was connected to a force sensor and pulled inside a 3D printed phantom with a well-defined geometry (Supplementary Fig. 30a). The channel has 6 turns each with 90° turn angle and is made of cured 3D printed resin (Supplementary Fig. 30b). The retraction forces were recorded while the μ-probe was pulled at 2 mm·s^{-1} under different flow rates.

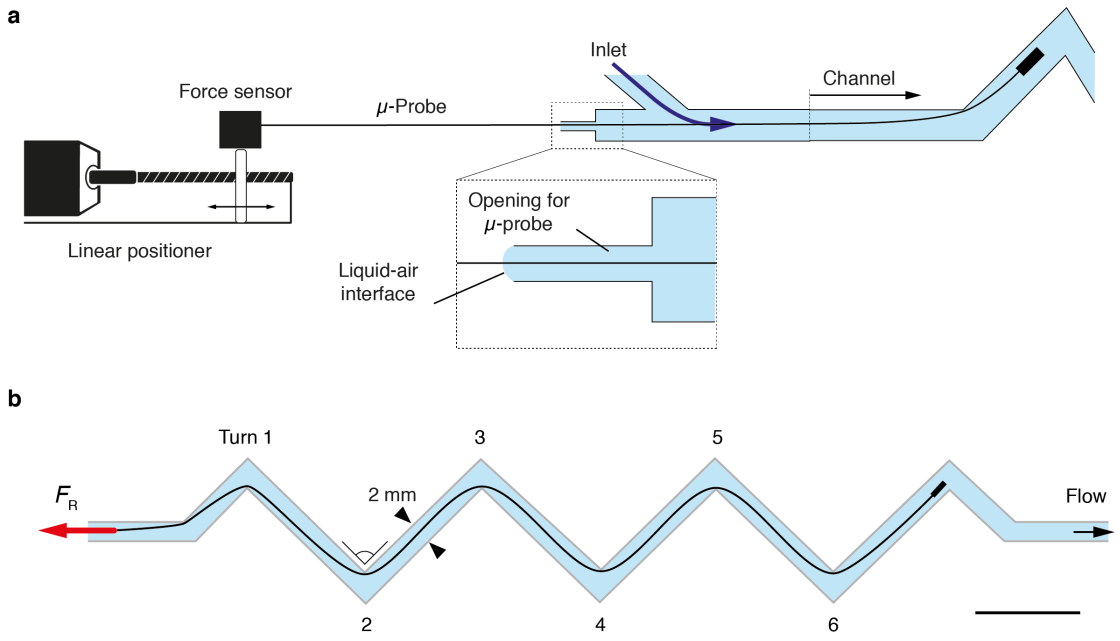

Supplementary Figure 30. **Measurement of the retraction force**. **a** Illustration showing the experimental setup, advancement mechanism, and the fluidic device. Drawings are not to scale. **b** The retraction forces (F_R) were measured in a phantom with 6 consecutives 90° turns under fluid flow. The μ-probe was initially placed at the end of the turn 6 and pulled back towards the entry point (to the left). Scale bar, 1 cm.

The experimental results are shown in Supplementary Figure 31. The retraction force increases exponentially with the turn number, as expected. We also see an increase in retraction forces with increasing flow velocity due to the increase in drag forces

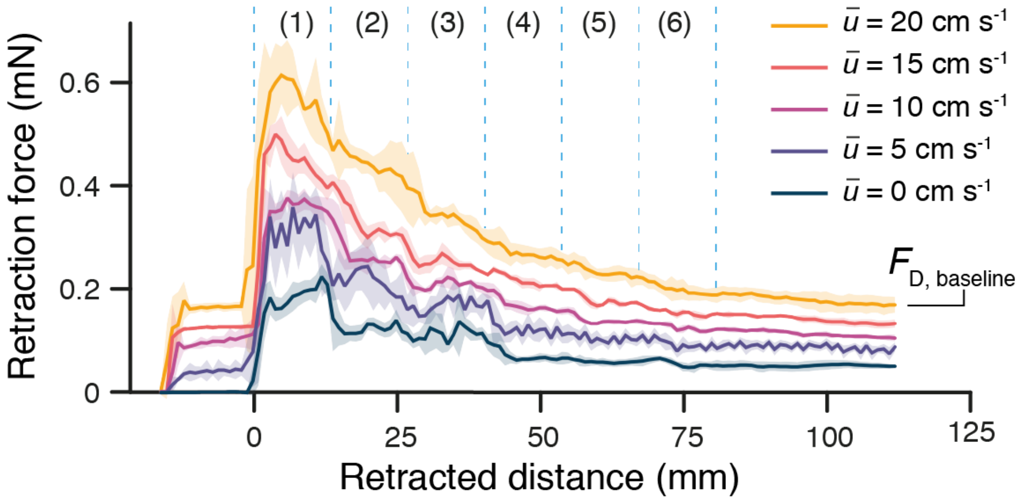

Supplementary Figure 31. Measurement of retraction forces in a channel with a series of 90° turns. Retraction forces recorded during the pulling of a µ-probe at 2 mm·s^{-1} at different flow rates. Regions (1) - (6) indicate the passage of the µ-probe through curved parts of the channel. Each test was repeated 5 times, darker lines represent the average and lighter areas represent the ± standard deviation.

We then normalized the data with respect to the baseline drag force, $F_{\mathrm{D,Baseline}}$. The normalized data essentially follow the same curve (Supplementary Fig. 32). Thus, the total force required to retract the µ-probe can be estimated for a given number of turns and the measured or calculated value of the drag force acting on the µ-probe just before the first turn.

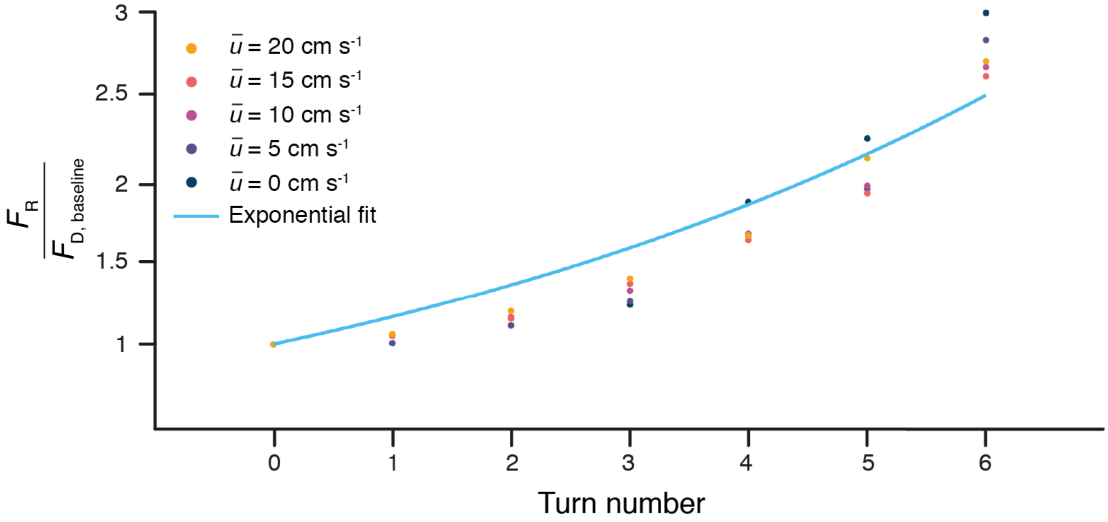

Supplementary Figure 32. Normalized retraction forces at different $\bar{u}$. The force required to retract the µ-probe as a function of the number of turns. The curves are normalized with respect to the baseline drag force. Blue curve represents the exponential fit for $\bar{u}$ = 20 cm·s^{-1}.

We next extracted the dynamic friction coefficient from the experimental data using the analytical formula. As the turn angle θ is kept constant at each turn, the total retraction force is calculated as:

$$F_{\mathrm{R}} = F_{\mathrm{D,Baseline}}\ (e^{\mu\theta})^{n} = F_{\mathrm{D,Baseline}}\ e^{n\mu\theta} \tag{34}$$

We used an exponential fit to the data recorded at $\bar{u}$ = 20 cm·s^{-1} to quantify the exponent of the exponential, which was found as 0.152. The dynamic friction coefficient is then found as:

$$\mu_{20} = \frac{0.152}{\theta} = \frac{0.152}{0.37\pi} = 0.13 \tag{35}$$

By repeating this calculation for different flow rates, the friction coefficient corresponding to different flow rates are found to be between 0.122 and 0.143. The difference may be related to a lubrication effect that is manifested with higher flow velocities. These values agree with the literature. For plastic-plastic friction, the reported values show μ smaller than 0.1 with lubrication and μ larger than 0.2 at dry conditions[7].

After this verification step, we can conclude that the total retraction force required to pull back a μ-probe that passes through *n* number of 90° turns, with dynamic friction coefficient $\mu_{20} = 0.13,$ can be calculated using the following simple formula:

$$F_{\mathrm{R}} = F_{\mathrm{D,Baseline}}\ e^{\mu_{20}\cdot 0.37\cdot\pi\cdot n} = F_{\mathrm{D,Baseline}}\ e^{0.152\,n} \tag{36}$$

In our phantoms filled with water, the baseline drag force is calculated as $F_{\mathrm{D,Baseline}}$ = 0.021 mN (see Supplementary Fig. 27b). For a μ-probe *l* = 5 cm, $\bar{u}$ = 5 cm·s^{-1}, and 20 turns, the total retraction force is

$$F_{\mathrm{R}} = F_{\mathrm{D,Baseline}}\ e^{0.152\,n} = 0.44\ \mathrm{mN} \tag{37}$$

We can calculate the retraction force under physiological conditions (*i.e.* blood viscosity and vessel friction) for 20 turns with the same μ-probe length and flow rate. The dynamic friction coefficient in the vessels is reported[8] as $\mu = 0.013$. Under these conditions, the baseline drag force

$$F_{\mathrm{D,Baseline}} = 0.041\ \mathrm{mN} \tag{38}$$

And

$$F_{\mathrm{Retraction}} = F_{\mathrm{D,Baseline}}\ e^{0.013\cdot 0.37\pi\cdot n} = 0.0423\ \mathrm{mN} \tag{39}$$

Despite the increased drag force provided by the blood, the ~10-fold decrease in dynamic friction coefficient leads to reduced forces. Likewise, the maximal normal force F_{N} and friction force (F_{F}) can be calculated:

$$F_{\mathrm{N}} = \frac{\boldsymbol{F}_{\mathrm{R}}}{\mu}\left[e^{\mu\theta} - 1\right] = \frac{0.0423}{0.013}\left[e^{0.013 \cdot 0.37\pi} - 1\right] = 0.0495\ \mathrm{mN} \tag{40}$$

$$F_{\mathrm{F}} = \mu F_{\mathrm{N}} = 0.64\ \mu\mathrm{N} \tag{41}$$

The total retraction force and the normal forces on the inward walls remain in the μN-range, assuring that no damage would occur during retraction, perforation, dissection, or otherwise.

Supplementary Note 5.3 During steering.

We analytically calculated the forces that are applied by the magnetic head due to application of magnetic fields using an experimentally validated model. We do not apply push or pull forces to the magnetic head because we use homogeneous magnetic fields for steering. The only force exerted by the magnetic head on vessel wall arise from the magnetic torque and the rotation of the head (Supplementary Fig. 33). These forces are transmitted at the longitudinal extremes of the head and are maximized with the application of a perpendicular magnetic field. We assumed the worst-case scenario in the following analysis to report the highest possible forces.

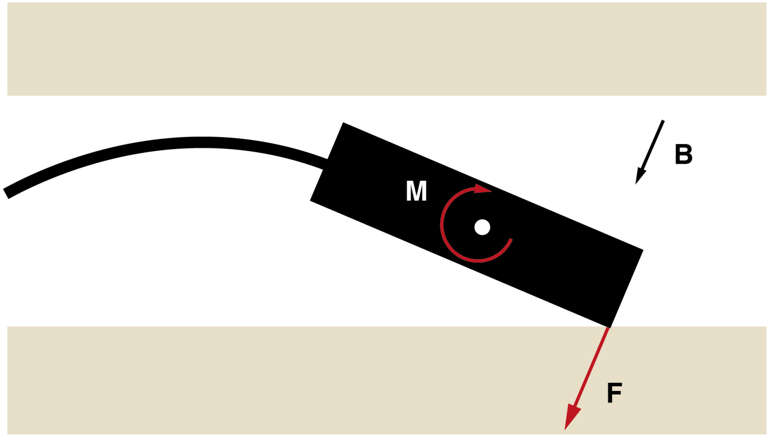

Supplementary Figure 33. Illustration showing the free body diagram of the magnetic head. Contact force was applied by the tip of the magnetic head on the vessel wall upon application of magnetic torque. Drawings are not to scale.

The total force acting at the tip can be computed from the torque – force relation assuming perpendicular actuation (*i.e.* magnetic field is perpendicular to the longitudinal axis of the μ-probe head):

$$F = \frac{M}{L} \tag{42}$$

where M is the torque applied by the magnetic field and L the distance between the centre of rotation and the tip. The magnetic torque is computed from:

$$\boldsymbol{M} = \boldsymbol{m} \times \boldsymbol{B} \tag{43}$$

where $\boldsymbol{m}$ is the magnetic dipole moment and $\boldsymbol{B}$ the applied magnetic field. The vector product imposes maximal torque at 90° between applied magnetic field and magnetization direction of the magnetic head ($\mathbf{B} \perp \mathbf{m}$). The magnetic dipole moment can be computed as:

$$m = \frac{1}{\mu_0} B_r V \tag{44}$$

where μ_0 is the permeability of vacuum ($= 4\pi \cdot 10^{-7}$ $H \cdot m^{-1}$), B_r is the factory-measured residual flux density of the neodymium iron boron (NdFeB) magnetic particles (0.8 T) and V is the volume of the magnetic head (20% volumetric ratio between NdFeB particles and PDMS).

Next, we calculated the maximal force that the magnetic head would exert on the vessel wall if we accidentally applied the magnetic field in the most misdirected way. The analytical results are shown in Supplementary Figure 34. The maximum field strength that we used for steering is 30 mT and the largest magnetic head has a diameter of 350 μm. For these parameters, the maximal force was calculated to be around 0.4 mN.

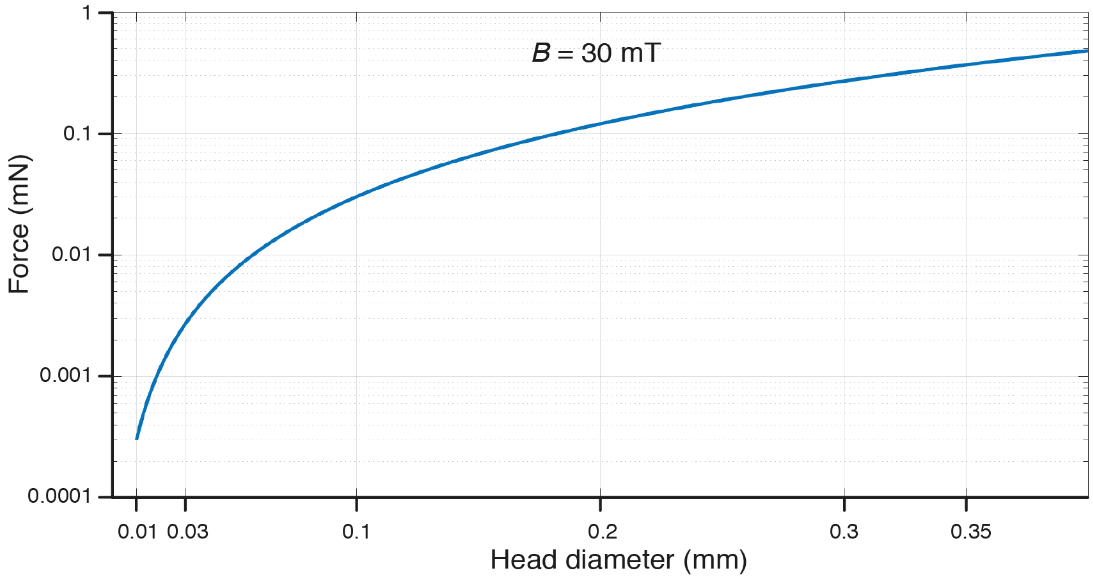

Supplementary Figure 34. **Contact force generated due to magnetic actuation**. Calculation of the magnitude of the contact force applied by the tip of the magnetic head on the vessel wall. The data is plotted as a function of the head diameter. Magnetic field is assumed to be perpendicular to the long axis of the head, at a constant magnitude of 30 mT.

We experimentally validated the analytical model by measuring the change in the weight of an actuated magnetic head (Supplementary Fig. 35). To avoid magnetic interaction between the Helmholtz coils and the scale, a light plastic bar was vertically positioned on the scale to transmit the forces applied from the fiber. We applied uniform magnetic field of 20 mT strength to a horizontally placed 5 mm long and 700 μm-thick magnetic fiber that was clamped at one end. For these parameters, the calculated force is 0.98 mN. The increase in mass that was measured by the scale was 0.09 g, which corresponds to 0.88 mN.

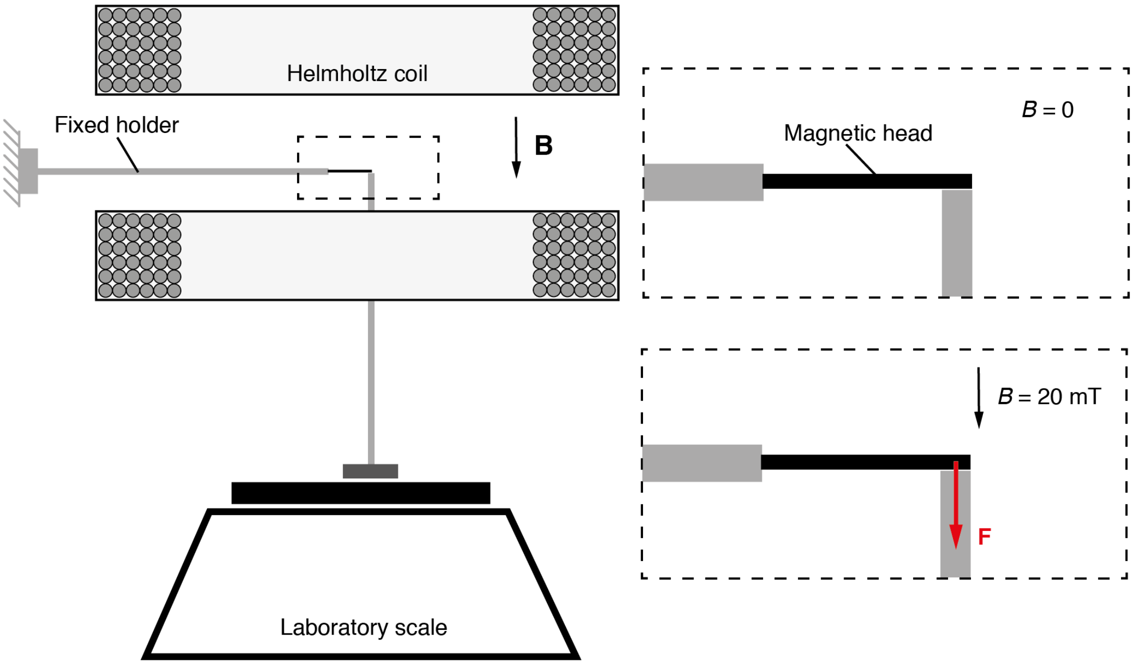

Supplementary Figure 35. **Schematics of the setup used to measure the force applied by the tip of the actuated magnetic head.** The setup consists of a pair of electromagnetic coils (Helmholtz configuration) placed above a sensitive scale. A lightweight bar serves as an adapter to transmit the forces generated by the clamped magnetic head that is kept in position by a fixed holder (top inset). Upon application of homogeneous magnetic field perpendicular to the long axis of the head, the structure rotates and presses on the vertical bar (bottom inset). Drawings are not to scale.

Supplementary Note 6. Fabrication of electronic µ-probes

The flow sensor has been fabricated by standard microfabrication techniques (Supplementary Fig. 36a). A silicon (Si) wafer, 4-inch, has been primed by oxygen (O_2) plasma for 5 min. A thin layer of polyimide (PI, PI2611 Hitachi Chemical DuPont MicroSystems GmbH, 3 µm) has been spin-coated at 4000 RPM for 30s. Curing of the polymer required an initial soft bake at 75 °C and at 115 °C for 3 min for each temperature, followed by a gradual hard bake. (3 °C·min^{-1} temperature ramp from room temperature to 200 °C under normal atmosphere, 1 h bake at 200 °C under N_2 atmosphere, 3 °C·min^{-1} temperature ramp to 300 °C and bake for 1 h under N_2 atmosphere and temperature decrease to 200 °C; room temperature restoration under normal atmosphere). After the PI surface activation by argon plasma, a titanium/platinum (Ti/Pt 10 nm/275 nm) adhesion/conductive layer was sputtered (Alliance Concept AC450). Patterning of the traces has been performed by spin-coating of positive photoresist (AZ9260, 8 µm), baking at 110 °C for 240 s, rehydrating at room temperature for 20 min followed by UV

exposure (Heidelberg Instruments MLA150, 405 nm and 340 mJ·cm^{-2}) and development (AZ400K for 360 s). Pt excess was removed by chlorine dry etching (Corial 210IL). Photoresist residuals have been removed by slow O_2 plasma for 1 min (Corial 210IL) and dipping for 5 min inside a Remover 1165 solution at 60 °C. To add the two serpentines, serving as temperature sensor and heater, another 25 nm-thick layer of platinum has been sputtered over the wafer, after surface activation by argon plasma (Alliance Concept AC450). To define the serpentines shapes a positive photoresist mask has been spin-coated (AZ1512, 2 µm) over the thin platinum layer, soft baked at 110 °C for 90 s, patterned by UV exposure (Heidelberg Instruments MLA150, 405 nm and 104 mJ·cm^{-2}) and developed (AZ 726 MIF developer for 45 s). Excess platinum was then dry etched with chlorine (Corial 210IL). To remove photoresist residual, O_2 plasma has been used for 1 min, the wafer has then been dipped in a Remover 1165 solution at 60 °C for 5 min. Before encapsulation of the device, an adhesion promoter (VM652) has been spin-coated. Encapsulating PI (PI2610 Hitachi Chemical DuPont MicroSystems GmbH, 1 µm) has been then deposited at 4000 RPM and cured by soft and hard bake (same process as above). Opening of the contacts has been done using a positive photoresist mask (AZ1512, 2 µm exposed by Heidelberg Instruments MLA150, 405 nm and 104 mJ·cm^{-2} and developed by AZ 726 MIF developer for 45 s) and PI etching by O_2 plasma (Corial 210IL). Final stripping of photoresist residuals has been carried out by dipping the wafer in a Remover 1165 solution at 60 °C for 5 min. The borders of the µ-probe have been then laser cut (Optec MM200-USP) to 300 µm x 65 mm rectangles and detachment from the wafer has been done manually.

Generic µ-probes for fluid-structure interactions studies have been prepared by spin-coating on a 4-inch Si wafer a 4 µm-thick layer of PI (PI2610 Hitachi Chemical DuPont MicroSystems GmbH) and baking with the same process as above (Supplementary Fig. 36b). A positive photoresist (AZ1512, 2 µm exposed by Heidelberg Instruments MLA150, 405 nm and 104 mJ·cm^{-2} and developed by AZ 726 MIF developer for 45 s) has been used as mask for sputtering (Alliance Concept AC450) a 100 nm-thick layer of gold in stripes of 250 µm-width

and 9 cm-length, followed by lift-off in acetone. The µ-probes borders have been then laser cut (Optec MM200-USP) and detachment from the wafer has been done manually. Finally, generic µ-probes without gold, have been fabricated as the latter without the gold deposition and laser-cutting stripes of different widths varying from 25 µm to 300 µm.

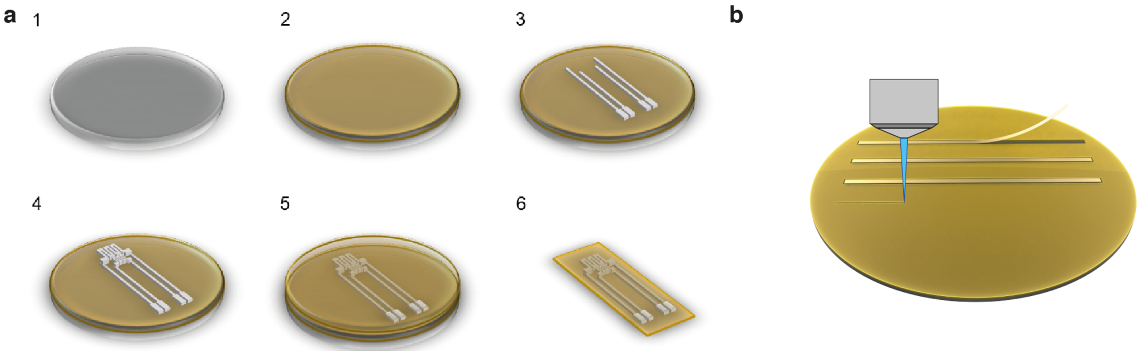

Supplementary Figure 36. Microfabrication of electronic µ-probes. **a** Microfabrication steps for the flow sensor: 1, a 4-inch Si wafer is prepared by O_2 plasma cleaning; 2, a 3 µm-thick polyimide layer is spin-coated and cured; 3, a first 275 nm-thick platinum layer is deposited and etched according to the traces profile of the heater and sensor elements; 4, a second 25 nm-thick layer of platinum is sputtered and etched according to the serpentine shapes of the heater and sensor elements; 5, the circuitry is encapsulated by spin-coating and curing of a 1 µm-thick layer of polyimide and openings for the connection pads have been etched; 6, the stripe containing the flow sensor is laser cut and manually detached from the wafer. **b** Generic µ-probes have been prepared by curing polyimide on a 4-inch Si wafer and laser cutting their shapes. Drawings are not to scale.

Supplementary References

1. Kunhappan, D., Harthong, B., Chareyre, B., Balarac, G. & Dumont, P. J. J. Numerical modeling of high aspect ratio flexible fibers in inertial flows. *Phys. Fluids* **29**, 093302 (2017).

2. Campanile, L. F. & Hasse, A. A simple and effective solution of the elastica problem. *Proc. Inst. Mech. Eng. Part C J. Mech. Eng. Sci.* **222**, 2513–2516 (2008).

3. Bruus, H. *Theoretical Microfluidics*. (OUP Oxford, 2008).

4. Rafii-Tari H., Payne C. J., Bicknell C., Kwok K. W. Cheshire N. J. W., Riga C., Yang G. Z. Objective assessment of endovascular navigation skills with force sensing. *Ann. Biomed. Eng.* **45**, 1315-1327 (2017).

5. Mokin M., *et al.* Assessment of distal access catheter performance during neuroendovascular procedures: measuring force in three-dimensional patient specific phantoms. *J. Neurointervent. Surg.* **11**, 619-622 (2019).

6. Lubarda V., The mechanics of belt friction revisited, *Int. J. Mech. Eng. Educ.* **42**, 97-112 (2014).

7. www.tribology-abc.com/abc/cof.htm

8. Takashima K., Shimomura R., Kitou T., Terada H., Yoshinaka K., Ikeuchi K. Contact and friction between catheter and blood vessel. *Tribol. Int.* **40**, 319-328 (2007).
