# Supplementary material for: Flow driven robotic navigation of microengineered endovascular probes
Source: Nat Commun. 2020 Dec 22;11:6356. doi: 10.1038/s41467-020-20195-z (PMC7755921; doi:10.1038/s41467-020-20195-z)
Supplement: Supplementary file 3 — Description of Additional Supplementary Files [file 41467_2020_20195_MOESM3_ESM.pdf]

Description of Additional Supplementary Files

File Name: Supplementary Movie 1
Description: Adaptive transport in tortuous channels. Unsupervised advancement of a µ-probe by fluidic stresses in a turtous channel.

File Name: Supplementary Movie 2
Description: Crucial role of flow. Demonstration showing the buckling of a µprobe that is pushed in the absence of flow. The motion of the ultraflexible structure is driven by the flow in the channel.

File Name: Supplementary Movie 3
Description: Numerical simulation of µ-probe transport. Simulation of µ-probe motion inside a tortuous channel.

File Name: Supplementary Movie 4
Description: Autonomous navigation in stenotic occlusions. Demonstration of autonomous obstacle avoidance and navigation in the presence of exegerrated stenotic conditions.

File Name: Supplementary Movie 5
Description: Magnetic field sweep. Influence of the applied magnetic field on the dynamics of the µ-probe pose under viscous flow.

File Name: Supplementary Movie 6
Description: Controlled-Lift (CL) navigation. Description of the CL navigation method for the steering of the µ-probe into the chosen target daughter vessel.

File Name: Supplementary Movie 7
Description: Controlled-Heading (CH) navigation. Description of the CH navigation method for the steering of the µ-probe into the chosen target daughter vessel.

File Name: Supplementary Movie 8
Description: Numerical analysis of CL and CH navigation. Simulation results showing the conceptual differences in daughter artery selection using CL or CH navigation methods.

File Name: Supplementary Movie 9
Description: Navigation in narrow channel. Navigation in channels slightly larger than the size of the probe head.

File Name: Supplementary Movie 10
Description: µ-Probe navigation in ex vivo perfused rabbit ear. Proof-ofconcept CH navigation inside perfused arterial microvasculature of ex vivo rabbit ear.

File Name: Supplementary Movie 11

Description: Navigation and dye injection in ex vivo perfused rabbit ear. Proof-of-concept navigation and injection of optical contrast liquid through a 40 µm-inner diameter ultraflexible magnetic µ-catheter inside an ex vivo perfused arterial microvasculature
